# State estimator based on an indirect Kalman filter for a hydraulically actuated multibody system

**DOI:** 10.1007/s11044-022-09814-3

**Published:** 2022-02-22

**Authors:** Suraj Jaiswal, Emilio Sanjurjo, Javier Cuadrado, Jussi Sopanen, Aki Mikkola

**Affiliations:** 1 Department of Mechanical Engineering, Lappeenranta University of Technology, Yliopistonkatu 34, 53850 Lappeenranta, Finland; 2 Laboratory of Mechanical Engineering, University of La Coruña, Escuela Politécnica Superior, Mendizábal s/n, 15403 Ferrol, Spain

**Keywords:** State estimator, State observer, Indirect Kalman filter, Multibody system dynamics, Hydraulic actuators, Monolithic simulation

## Abstract

In multibody system dynamics, the equations of motion are often coupled with systems of other physical nature, such as hydraulics. To infer the real dynamical state of such a coupled multibody system at any instant of time, information fusing techniques, such as state estimators, can be followed. In this procedure, data is combined from the coupled multibody model and the physical sensors installed on the actual machine. This paper proposes a novel state estimator developed by combining a multibody model with an indirect Kalman filter in the framework of hydraulically driven systems. An indirect Kalman filter that utilizes the exact Jacobian matrix of the plant at position and velocity level is extended for hydraulically actuated systems. The structures of the covariance matrices of the plant and measurement noise are also studied. The multibody system, described using a semi-recursive formulation, and the hydraulic subsystem, described using lumped fluid theory, are coupled using a monolithic approach. As a case study, the state estimator is applied to a hydraulically actuated four-bar mechanism. The state estimator considers modeling errors in the force model because of its uncertainty in modeling. The measurements are obtained from a dynamic model which is considered as the ground truth, with an addition of white Gaussian noise to represent the noise properties of the actual sensors. The state estimator uses four sensor configurations with different sampling rates. For the presented case study, the state estimator can accurately estimate the work cycle and hydraulic pressures of the coupled multibody system. The results demonstrate the efficacy of the proposed state estimator.

## Introduction

Computer simulation of mechanical systems can be carried out using multibody system dynamics in which the equations of motion describe an equilibrium for the dynamic system under consideration. In multibody system dynamics, the equations of motion are often coupled with systems of other physical nature, such as hydraulic actuators and electric drives. If the forces are accurately defined, then such a coupled multibody system can accurately describe reality. Nevertheless, even such accurate models can deviate from reality over a long simulation run because of the accumulation of small modeling errors over time. To infer the real dynamical state of a multibody system at any instant of time, information fusing techniques, such as state estimators or observers, can be followed to combine data from the coupled multibody model and the real sensors installed on the actual machine. A state estimator, in general, is a recursive Bayesian estimator [33]. It can find its application to control and monitor the operation of the machine [22] by estimating the state subject of control instead of measuring it. In many practical cases, measurement can be cumbersome or expensive to conduct.

In mobile working machinery, the use of the multibody system approach is often combined with a description of the hydraulic actuators. Modeling of hydraulics often leads to numerically stiff systems [24, 29]. For such systems, the efficiency requirements in real-time applications are high [15, 16]. The problem of numerical stiffness can be alleviated by a proper selection of the multibody approach, as shown in [17]. Furthermore, to control and monitor the operation of a mobile working machine, it may become cumbersome or expensive to obtain the measurements of all the required physical quantities. For example, it may not be feasible or possible to measure the hydraulic forces acting on the multibody system in such an application. In this situation, state estimators can provide the possibility of using virtual sensors to infer measurements from the readings of different sensors, which are more economical or easy to install and maintain than the replaced one.

In recent years, there have been a number of studies on multibody-based state estimators. As a consequence, a number of estimation techniques such as Kalman filters [13, 33] and particle filters [1, 6] have been used in the literature [4, 9]. The application of Kalman filters to general multibody systems is not a trivial task because of their different mathematical structures. The Kalman filter was originally formulated for the first order linear and unconstrained systems [21]. Multibody dynamic systems are, in general, second order nonlinear and constrained systems [20]. Therefore, it is an open field for additional studies in general and more specifically for the study of state estimators for multibody systems, which include hydraulic actuators [17, 29].

The methods within the family of non-linear Kalman filters can be, in general, categorized into two groups, namely, independent coordinate methods and dependent coordinate methods [33]. In independent coordinate methods, the state vector of the filter comprises the independent coordinates only, which is the case for most probabilistic estimators presented in the estimation and control theory literature [13]. In this approach, the usual way to combine a multibody system with a Kalman filter is to use the independent coordinates and velocities of the multibody system as the state vector of the filter [35]. On the other hand, dependent coordinate methods [12, 34] can handle the case of intra-state vector dependencies, which is exactly the situation found in multibody dynamic systems. This approach can be applied to a multibody system by incorporating the constraints as perfect measurements or by projecting the unconstrained estimation over the constraints manifold [33]. For simplicity, only methods based on independent coordinates are covered in this work.

Among the methods in independent coordinates, a continuous extended Kalman filter (CEKF) was used to demonstrate a real-time state estimator for a four-bar mechanism [9]. In CEKF, the dynamic multibody equations are adapted to the structure of the Kalman filter in a continuous-time frame so that the state-transition and state-update stages are seamlessly fused together. In the rest of the estimators that work in discrete time steps, such as the discrete extended Kalman filter (DEKF) [35], the unscented Kalman filter (UKF) [25], and indirect Kalman filter [31], the filter formulation considers these stages as two separate steps. Both stages constitute different equations for updating the state vector and the associated covariance matrix. In these formulations, the state-transition or prediction stage relies on the transition model of the dynamic system, while the state-update stage includes information from the sensors or observations [35].

In [32], the accuracy and efficiency of CEKF, DEKF, UKF and indirect Kalman filter applied to four-bar and five-bar mechanisms, were compared. The indirect Kalman filter outperformed the others and was less affected by the increase in the size of the system. It even finds its application in automobiles [26] with real-time capability [30]. In an indirect Kalman filter, the position and velocity errors of the independent coordinates of a dynamic system are considered as the state vector of the filter. Thus, it is also referred to as the error-state extended Kalman filter (eEKF). However, this method assumes a simplified form of the transition model of the dynamic system, that is, the Jacobian matrix of its plant. This can lead to incorrect estimation in certain examples, as presented in [32]. Nevertheless, this short-come of eEKF has been addressed in [31] using an exact Jacobian matrix of the plant, referred to as the eEKF-EJ method. In [31], another variant of eEKF was proposed that additionally considers the acceleration errors of the independent coordinates in the state vector. This variant of eEKF can estimate the input force for the system and by its nature provides better accuracy than the variants without force estimation. Note that the indirect Kalman filter and its variants are independent of the type of integrator used in the dynamic system.

Despite the previous research efforts explained above, the limitations in the existing literature are twofold. First, even though there are various studies on multibody-based state estimators, their application in the field of hydraulically actuated multibody systems has been overlooked. Second, even though the literature on hydraulic machinery covers various aspects of modeling and simulation, their detailed investigation using extended or unscented Kalman filters has been neglected. Therefore, this study claims to cover this research gap by introducing a state estimator based on an indirect Kalman filter for hydraulically actuated multibody systems.

The objective of this paper is to propose a novel state estimator developed by combining a multibody model with an indirect Kalman filter in the framework of hydraulically driven systems. The eEKF-EJ method that utilizes the exact Jacobian matrix of the plant at position and velocity level, introduced in [31], is extended for hydraulically actuated systems. To complement the above objective, the structures of the covariance matrices of the plant and measurement noise are also explained. The multibody system, described using a semi-recursive formulation [2, 8, 10], and the hydraulic subsystem, described using the lumped fluid theory [37], are combined using a monolithic approach. As a case study, the state estimator is applied to a hydraulically actuated four-bar mechanism. Although a case study of a planar mechanism is shown in this study, the method presented is general and applicable to three-dimensional mechanisms as well. The state estimator considers modeling errors in the force model because of its uncertainty in modeling [31]. Measurements are obtained from a dynamic model, which is considered as a ground truth, with an addition of white Gaussian noise, to represent the noise properties of the actual sensors. The state estimator considers four sensor configurations at four different sampling rates of the sensors. For the presented case study, the state estimator is evaluated based on the accuracy of the work cycle and hydraulic pressures.

## Multibody formulation

In this study, the dynamics of a constrained mechanical system is described using a semi-recursive formulation. In this formulation, the motion of a system is described using the dynamics of the open-loop system and then incorporating the loop-closure constraints by means of the penalty-based approach [2, 8, 10]. The dynamics of the hydraulic actuators are modeled using the lumped fluid theory [37] and coupled with the dynamics of the multibody system in a monolithic approach. It should be noted that the hydraulic actuators are not considered as separate bodies, instead, the hydraulic forces are computed and fed into the multibody system as external forces. In this study, a semi-recursive formulation and lumped fluid theory are considered because they lead to a computationally efficient method for a unified simulation of multibody and hydraulic dynamics as presented in [28], [29] and [17].

### Semi-recursive formulation

Consider a system as an open-loop system with ${N_{b}}$ bodies, which may require temporary cutting of certain joints. The Cartesian velocities ${\mathbf{{Z}}}_{j} \in \mathbb{R}^{6 \times 1}$ and the Cartesian accelerations ${\dot{\mathbf{{Z}}}}_{j} \in \mathbb{R}^{6 \times 1}$ of a reference point on body $j$ can be written as ${\mathbf{{Z}}}_{j} = \left [ {\dot{\mathbf{{s}}}}_{j}^{\text{T}}, { \boldsymbol{\upomega }}_{j}^{\text{T}} \right ]^{\text{T}}$ and ${\dot{\mathbf{{Z}}}}_{j} = \left [ {\ddot{\mathbf{{s}}}}_{j}^{\text{T}}, { \dot{\boldsymbol{\upomega }}}_{j}^{\text{T}} \right ]^{\text{T}}$. Here, ${\dot{\mathbf{{s}}}}_{j}$ and ${\ddot{\mathbf{{s}}}}_{j}$ are, respectively, the velocity and the acceleration of the reference point attached to body ${j}$ that instantaneously coincides with the origin of the inertial reference frame, and ${\boldsymbol{\upomega }}_{j}$ and ${\dot{\boldsymbol{\upomega }}}_{j}$ are, respectively, the angular velocity and angular acceleration of the body ${j}$. The Cartesian velocities, ${\mathbf{{Z}}}_{j}$, and the Cartesian accelerations, ${\dot{\mathbf{{Z}}}}_{j}$, can be recursively expressed in terms of the previous bodies as [19]:

1 $$ {\mathbf{{Z}}}_{j} = {\mathbf{{Z}}}_{j-1} + {\mathbf{{b}}}_{j} {\dot{z}}_{j} $$

2 $$ {\dot{\mathbf{{Z}}}}_{j} = {\dot{\mathbf{{Z}}}}_{j-1} + {\mathbf{{b}}}_{j} {\ddot{z}}_{j} + {\mathbf{{d}}}_{j} $$

where the scalars ${\dot{z}}_{j}$ and ${\ddot{z}}_{j}$ are, respectively, the time and double time derivatives of the relative joint coordinate ${z}_{j}$, and the vectors ${\mathbf{{b}}}_{j}$ and ${\mathbf{{d}}}_{j}$ depend on the type of joint [11] that connects the bodies ${j-1}$ and ${j}$. The vector ${\mathbf{{b}}}_{j}$ are the Cartesian velocities ${\mathbf{{Z}}}_{j}$ of body ${j}$ when all relative joint velocities are made zero except for ${\dot{z}}_{j} = 1$; and the vector ${\mathbf{{d}}}_{j}$ is the difference of the Cartesian accelerations ${\dot{\mathbf{{Z}}}}_{j} - {\dot{\mathbf{{Z}}}}_{j-1}$ when all the relative joint accelerations are made zero [7, 8]. Note that the indexes $j-1$ and $j$ may not be successive as the system may branch. The mapping of the vector of Cartesian velocities, ${\mathbf{{Z}}} = \left [ {\mathbf{{Z}}}_{1}^{\text{T}}, {\mathbf{{Z}}}_{2}^{\text{T}}, \ldots , {\mathbf{{Z}}}_{N_{b}}^{\text{T}} \right ]^{\text{T}}$, into the vector of relative joint velocities, ${\dot{\mathbf{{z}}}} = \left [ {\dot{z}}_{1}, {\dot{z}}_{2}, \ldots , { \dot{z}}_{N_{b}} \right ]^{\text{T}}$, can be achieved using a velocity transformation matrix ${\mathbf{{R}}} \in {\mathbb{R}}^{6 {N_{b}} \times {N_{b}}}$ as [11, 19]:

3 $$ {\mathbf{{Z}}} = {\mathbf{{R}}} {\dot{\mathbf{{z}}}} = {\mathbf{{T}}} {\mathbf{{R}}}_{\text{d}} { \dot{\mathbf{{z}}}} $$

4 $$ {\dot{\mathbf{{Z}}}} = {\mathbf{{R}}} {\ddot{\mathbf{{z}}}} + {\dot{\mathbf{{R}}}} { \dot{\mathbf{{z}}}} = {\mathbf{{T}}} {\mathbf{{R}}}_{\text{d}} {\ddot{\mathbf{{z}}}} + {\mathbf{{T}}} {\dot{\mathbf{{R}}}}_{\text{d}} {\dot{\mathbf{{z}}}} $$

where ${\mathbf{{T}}} \in {\mathbb{R}}^{6 {N_{b}} \times 6 {N_{b}}}$ is the constant path matrix that describes the topology of the open-loop system, and ${\mathbf{{R}}}_{\text{d}} \in {\mathbb{R}}^{6 {N_{b}} \times {N_{b}}}$ is a diagonal matrix whose elements are the vectors ${\mathbf{{b}}}_{j}$ arranged in an ascending order. Note that the term ${\dot{\mathbf{{R}}}} {\dot{\mathbf{{z}}}}$ in Eq. (4) can be expressed in terms of the vectors ${\mathbf{{d}}}_{j}$ using Eq. (2) [11].

Using the principle of virtual work, the equations of motion for the open-loop system can be written as [19]:

5 $$ {\mathbf{{R}}}_{\text{d}}^{\text{T}} {\mathbf{{T}}}^{\text{T}} {\bar{\mathbf{{M}}}} {\mathbf{{T}}} {\mathbf{{R}}}_{\text{d}} {\ddot{\mathbf{{z}}}} = {\mathbf{{R}}}_{\text{d}}^{\text{T}} { \mathbf{{T}}}^{\text{T}} \left ( {\bar{\mathbf{{Q}}}} - {\bar{\mathbf{{M}}}} {\mathbf{{T}}} { \dot{\mathbf{{R}}}}_{\text{d}} {\dot{\mathbf{{z}}}} \right ) $$

where ${\ddot{\mathbf{{z}}}} \in {\mathbb{R}}^{N_{b}}$ is the vector of relative joint accelerations, and the matrices ${\bar{\mathbf{{M}}}} \in {\mathbb{R}}^{6 {N_{b}} \times 6 {N_{b}}}$ and ${\bar{\mathbf{{Q}}}} \in {\mathbb{R}}^{6 {N_{b}}}$ are, respectively, a diagonal matrix that consists of the mass matrices ${\bar{\mathbf{{M}}}}_{j}$ and a column vector that consists of the force vectors ${\bar{\mathbf{{Q}}}}_{j}$ such that:

6 $$ {\bar{\mathbf{{M}}}}_{j} = \begin{bmatrix} {m_{j}} {\mathbf{{I}}}_{3} & - {m_{j}} {\widetilde{\mathbf{{g}}}}_{j} \\ {m_{j}} {\widetilde{\mathbf{{g}}}}_{j} & {\mathbf{{J}}}_{j} - {m_{j}} { \widetilde{\mathbf{{g}}}}_{j} {\widetilde{\mathbf{{g}}}}_{j} \end{bmatrix} $$

7 $$ \begin{aligned} {\bar{\mathbf{{Q}}}}_{j} = \begin{bmatrix} {\mathbf{{f}}}_{j} - {\widetilde{\boldsymbol{\upomega }}}_{j} \left ( { \widetilde{\boldsymbol{\upomega }}}_{j} {m_{j}} {\mathbf{{g}}}_{j}\right ) \\ {\boldsymbol{\uptau }}_{j} - {\widetilde{\boldsymbol{\upomega }}}_{j} { \mathbf{{J}}}_{j} {\boldsymbol{\upomega }}_{j} + {\widetilde{\mathbf{{g}}}}_{j} \left ( {\mathbf{{f}}}_{j} - {\widetilde{\boldsymbol{\upomega }}}_{j} \left ( {\widetilde{\boldsymbol{\upomega }}}_{j} {m_{j}} {\mathbf{{g}}}_{j}\right ) \right ) \end{bmatrix} \end{aligned} $$

where ${m_{j}}$ is the mass of body $j$, ${\mathbf{{I}}}_{3}$ is a ${\left (3 \times 3\right )}$ identity matrix, ${\mathbf{{g}}}_{j}$ is the position vector of the centre of mass of body $j$, the skew-symmetric matrix of a vector is denoted by a tilde (~), ${\mathbf{{J}}}_{j}$ is the inertia tensor of body $j$, ${\mathbf{{f}}}_{j}$ is the vector of external forces applied on body $j$, and ${\boldsymbol{\uptau }}_{j}$ is the vector of external moments with respect to the center of mass of body $j$.

**Fig. 1:**
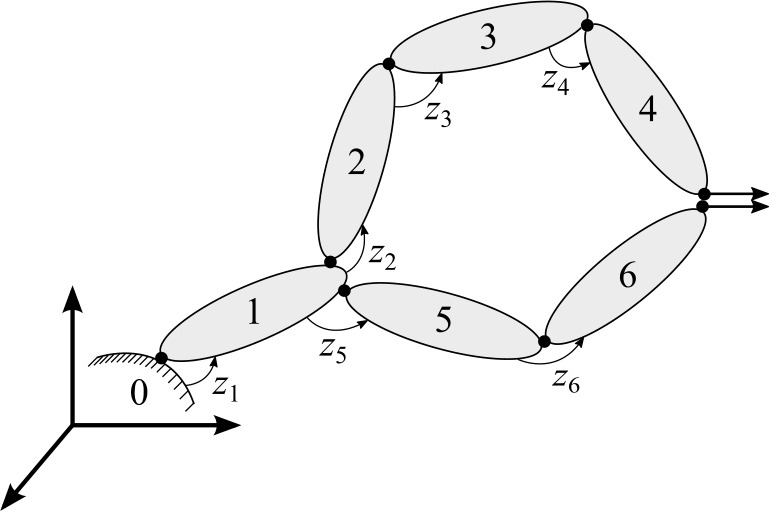
An example of a closed-loop system

The equations of motion for the closed-loop system can be written by incorporating a set of $m$ loop-closure constraint equations, ${\boldsymbol{\Phi }} = \mathbf{{0}}$, in Eq. (5) as [10, 27]:

8 $$ {\mathbf{{M}}} {\ddot{\mathbf{{z}}}} + {\boldsymbol{\Phi }}_{\mathbf{{z}}}^{\text{T}} \alpha {\boldsymbol{{ \Phi }}} + {\boldsymbol{\Phi }}_{\mathbf{{z}}}^{\text{T}} {\boldsymbol{\uplambda }} = { \mathbf{{Q}}} $$

where ${\boldsymbol{\Phi }}_{\mathbf{{z}}}$ is the Jacobian matrix of ${\boldsymbol{\Phi }} \left ( {\mathbf{{z}}} \right ) = \mathbf{{0}}$, $\alpha $ is the penalty factor that can be set the same for all constraints, ${\boldsymbol{\uplambda }}$ is the vector of iterated Lagrange multipliers, ${\mathbf{{M}}} = \left ( {\mathbf{{R}}}_{\text{d}}^{\text{T}} {\mathbf{{T}}}^{\text{T}} { \bar{\mathbf{{M}}}} {\mathbf{{T}}} {\mathbf{{R}}}_{\text{d}} \right )$, and ${\mathbf{{Q}}} = \left [ {\mathbf{{R}}}_{\text{d}}^{\text{T}} {\mathbf{{T}}}^{\text{T}} \left ( {\bar{\mathbf{{Q}}}} - {\bar{\mathbf{{M}}}} {\mathbf{{T}}} {\dot{\mathbf{{R}}}}_{ \text{d}} {\dot{\mathbf{{z}}}} \right ) \right ]$. Note that for simplicity, the constraint equations are assumed to be holonomic and scleronomic. In this formulation, ${\boldsymbol{\uplambda }}$ are iterated at each time-step $k$ as [10]:

9 $$ {\boldsymbol{\uplambda }}_{k}^{(h+1)} = {\boldsymbol{\uplambda }}_{k}^{(h)} + \alpha {\boldsymbol{\Phi }}_{k}^{(h+1)} $$

where $h$ is the iteration step and ${\boldsymbol{\uplambda }}_{k}^{(0)}$ is the final value of ${\boldsymbol{\uplambda }}_{k-1}$ from the previous time-step. Figure 1 shows an example of a closed-loop system.

The system is integrated using the implicit single-step trapezoidal rule [10]. In this formulation, the relative joint velocities, ${\dot{\mathbf{{z}}}}$, and accelerations, ${\ddot{\mathbf{{z}}}}$, are corrected using the mass-damping-stiffness-orthogonal projections as [3, 8]:

10 $$ \left [ {\mathbf{{W}}} + {\frac{\Delta t^{2}}{4}} {\boldsymbol{\Phi }}_{\mathbf{{z}}}^{ \text{T}} \alpha {\boldsymbol{\Phi }}_{\mathbf{{z}}} \right ] {\dot{\mathbf{{z}}}} = {\mathbf{{W}}} {\dot{\mathbf{{z}}}}^{\prime } - {\frac{\Delta t^{2}}{4}} {\boldsymbol{\Phi }}_{\mathbf{{z}}}^{ \text{T}} \alpha {\boldsymbol{\Phi }}_{t} $$

11 $$ \left [ {\mathbf{{W}}} + {\frac{\Delta t^{2}}{4}} {\boldsymbol{\Phi }}_{\mathbf{{z}}}^{ \text{T}} \alpha {\boldsymbol{\Phi }}_{\mathbf{{z}}} \right ] {\ddot{\mathbf{{z}}}} = {\mathbf{{W}}} {\ddot{\mathbf{{z}}}}^{\prime } - {\frac{\Delta t^{2}}{4}} {\boldsymbol{\Phi }}_{\mathbf{{z}}}^{ \text{T}} \alpha \left ( {\dot{\boldsymbol{\Phi }}}_{\mathbf{{z}}} {\dot{\mathbf{{z}}}} + { \dot{\boldsymbol{\Phi }}}_{t} \right ) $$

where ${\dot{\mathbf{{z}}}}^{\prime }$ and ${\ddot{\mathbf{{z}}}}^{\prime }$ are, respectively, the relative joint velocities and accelerations obtained from the Newton–Raphson iteration, $\Delta t$ is the time-step, ${\mathbf{{W}}} = {\mathbf{{M}}} + {\frac{\Delta t}{2}}{\mathbf{{C}}} + { \frac{\Delta t^{2}}{4}}{\mathbf{{K}}}$, where ${\mathbf{{C}}}$ and ${\mathbf{{K}}}$ represent the damping and stiffness contributions in the system, ${\boldsymbol{\Phi }}_{t}$ is the partial derivative of the constraints with respect to time $t$, and the term $\left ( {\dot{\boldsymbol{\Phi }}}_{\mathbf{{z}}} {\dot{\mathbf{{z}}}} \right )$ can be calculated from the chain rule of differentiation by using as intermediate variables, the coordinates of points and the components of unit vectors, as those shown in Fig. 1.

### Hydraulic actuators

In this study, hydraulic pressures within a hydraulic circuit are described using the lumped fluid theory [37]. According to the lumped fluid theory, a hydraulic circuit can be divided into discrete volumes, where pressures are assumed to be evenly distributed. Thus, the effects of acoustic waves are ignored. The pressure build-up, $\dot{p}_{s}$, in a hydraulic section $S$ can be expressed as:

12 $$ \dot{p}_{s} = \frac{B_{e_{s}}}{V_{s}} \sum _{k=1}^{n_{f}} {Q_{s k}} $$

where ${V_{s}}$ is the volume of the section, ${B_{e_{s}}}$ is its effective bulk modulus, ${Q_{s k}}$ is the incoming or outgoing flows of the section, and ${n_{f}}$ is the total number of volume flows going in or out of the section. In Eq. (12), ${B_{e_{s}}}$ can be written as:

13 $$ {B_{e_{s}}} = \left (\frac{1}{B_{oil}} + \sum _{k=1}^{n_{c}} \frac{V_{k}}{{V_{s}}{B_{k}}} \right )^{-1} $$

where ${B_{oil}}$ is the bulk modulus of oil, ${B_{k}}$ is the bulk modulus of volume ${V_{k}}$, and ${n_{c}}$ is the total number of sub-volumes ${V_{k}}$, such as pipes and hoses, that form the volume ${V_{s}}$.

#### Valves

The valves in a hydraulic circuit can be described using a semi-empirical modeling method [14]. In this method, the volume flow rate, ${Q_{t}}$, through a simple throttle valve can be expressed as:

14 $$ {Q_{t}} = {C_{v_{t}}} \ {{\text{sgn}}({\Delta p})} \ {\sqrt{\mid \Delta p \mid }} $$

where ${C_{v_{t}}}$ is the semi-empirical flow rate coefficient of the throttle valve and ${\Delta p}$ is the pressure difference over the valve. In Eq. (14), ${C_{v_{t}}}$ can be written as:

15 $$ {C_{v_{t}}} = {C_{d}} {A_{t}} \sqrt{\frac{2}{\rho }} $$

where ${A_{t}}$ is the area of the throttle valve, ${C_{d}}$ is its flow discharge coefficient and ${\rho }$ is the oil density. The value of ${C_{d}}$ can range between 0 and 1, signifying how much the actual flow differs from a reference flow for the same restriction and geometry of the valve. In this study, the value of ${C_{d}}$ is considered to be 0.8, as in [17, 29].

Similarly, the volume flow rate, ${Q_{d}}$, through a directional control valve can be expressed as:

16 $$ {Q_{d}} = {C_{v_{d}}} {U} \ {{\text{sgn}}({\Delta p})} \ {\sqrt{\mid \Delta p \mid }} $$

where ${C_{v_{d}}}$ is the semi-empirical flow rate constant of the directional control valve that can be procured from manufacturers’ catalogues, and ${U}$ is the relative spool position that can be computed as:

17 $$ \dot{U} = \frac{{U_{ref}} - {U}}{\tau } $$

where ${U_{ref}}$ is the reference voltage signal for the reference spool position and ${\tau }$ is the time constant, which can be procured from the Bode-diagram of the valve that describes the valve spool dynamics. In this study, the value of ${C_{v_{d}}}$ is computed for a nominal flow (flow at full opening) of 24 l/min over a 35 bar pressure difference. Note that for a pressure difference of less than 2 bar, the volume flow is assumed laminar, and Eqs. (14) and (16) are modified so that the volume flow and the pressure difference follow a linear relation.

#### Hydraulic cylinders

**Fig. 2:**
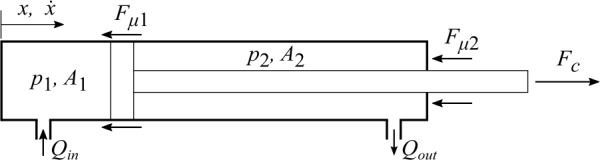
Schematic figure of a hydraulic cylinder

A hydraulic cylinder is shown in Fig. 2, whose motion produces volume flows that can be written as:

18 $$ {Q_{in}} = {\dot{x}}{A_{1}} , \quad {Q_{out}} = {\dot{x}}{A_{2}} $$

where ${Q_{in}}$ and ${Q_{out}}$ are, respectively, the incoming and outgoing volume flow rates of the hydraulic cylinder, ${A_{1}}$ and ${A_{2}}$ are, respectively, the piston and piston-rod side areas of the cylinder, and ${\dot{x}}$ is the piston velocity. The force ${F_{c}}$ produced by the cylinder can be expressed as:

19 $$ {F_{c}} = {p_{1}}{A_{1}} - {p_{2}}{A_{2}} - {F_{\mu }} $$

where ${p_{1}}$ and ${p_{2}}$ are, respectively, the pressures on the piston and piston-rod sides, and ${F_{\mu }}$ is the total friction force [5] caused by the seal. The friction force from sealing can be computed using various friction models as presented by the authors in [18]. In this study, a continuous static friction model, namely, Brown-McPhee friction model [5], is utilized because it can describe the usual friction characteristics with a computationally efficient approach, as presented by the authors in [18].

### Coupling of multibody system and hydraulic actuators

The multibody system, described in Sect. 2.1, is extended to incorporate the dynamics of the hydraulic actuators, described in Sect. 2.2, in a monolithic approach [17, 24, 29]. The force vector, ${\mathbf{{Q}}}$, in Eq. (8) is extended with the pressure variation equations, which can lead to a combined system of equations as in [29]:

20 $$ \left . \begin{aligned} {\mathbf{{M}}} {\ddot{\mathbf{{z}}}} + {\boldsymbol{\Phi }}_{\mathbf{{z}}}^{\text{T}} \alpha {\boldsymbol{{\Phi }}} + {\boldsymbol{\Phi }}_{\mathbf{{z}}}^{\text{T}} {\boldsymbol{\uplambda }} &= { \mathbf{{Q}}} \left ( {\mathbf{{z}}}, {\dot{\mathbf{{z}}}}, {\mathbf{{p}}} \right ) \\ {\dot{\mathbf{{p}}}} &= {\mathbf{{v}}} \left ( {\mathbf{{p}}}, {\mathbf{{z}}}, {\dot{\mathbf{{z}}}} \right ) \end{aligned} \, \right \} $$

where ${\dot{\mathbf{{p}}}}$ is the time derivative of the vector of pressures, ${\mathbf{{p}}}$, in the hydraulic subsystem, and ${{\mathbf{{v}}} \left ( {\mathbf{{p}}}, {\mathbf{{z}}}, {\dot{\mathbf{{z}}}} \right )}$ are the pressure variation equations. The dependencies of ${\mathbf{{Q}}}$ and the function ${\mathbf{{v}}}$ with respect to ${\mathbf{{z}}}$, ${\dot{\mathbf{{z}}}}$, and ${\mathbf{{p}}}$ are assumed to be known.

In this study, the coupled system is integrated using the implicit single-step trapezoidal rule [23]. By initially applying the predictors, ${{\mathbf{{z}}}_{k+1} = {\mathbf{{z}}}_{k} + {\dot{\mathbf{{z}}}}_{k} \Delta t + \frac{1}{2} {\ddot{\mathbf{{z}}}}_{k} \Delta t^{2}}$ and ${{\mathbf{{p}}}_{k+1} = {\mathbf{{p}}}_{k} + {\dot{\mathbf{{p}}}}_{k} \Delta t }$, respectively, to the relative joint coordinates, ${\mathbf{{z}}}_{k+1}$, and the pressures, ${\mathbf{{p}}}_{k+1}$, the solution for the relative joint velocities, ${\dot{\mathbf{{z}}}}_{k+1}$, the relative joint accelerations, ${\ddot{\mathbf{{z}}}}_{k+1}$, and pressure derivatives, ${\dot{\mathbf{{p}}}}_{k+1}$, at time-step $\left ( k+1 \right )$ can, respectively, be written as:

21 $$ \left . \begin{aligned} {\dot{\mathbf{{z}}}}_{k+1} &= \frac{2}{\Delta t} {\mathbf{{z}}}_{k+1} + { \check{\dot{\mathbf{{z}}}}}_{k} \\ {\ddot{\mathbf{{z}}}}_{k+1} &= \frac{4}{\Delta t^{2}} {\mathbf{{z}}}_{k+1} + { \check{\ddot{\mathbf{{z}}}}}_{k} \\ {\dot{\mathbf{{p}}}}_{k+1} &= \frac{2}{\Delta t} {\mathbf{{p}}}_{k+1} + { \check{\dot{\mathbf{{p}}}}}_{k} \end{aligned} \, \right \} $$

where

22 $$ \left . \begin{aligned} {\check{\dot{\mathbf{{z}}}}}_{k} &= - \left ( \frac{2}{\Delta t} {\mathbf{{z}}}_{k} + \dot{\mathbf{{z}}}_{k} \right ) \\ {\check{\ddot{\mathbf{{z}}}}}_{k} &= - \left ( \frac{4}{\Delta t^{2}} {\mathbf{{z}}}_{k} + \frac{4}{\Delta t} \dot{\mathbf{{z}}}_{k} + \ddot{\mathbf{{z}}}_{k} \right ) \\ {\check{\dot{\mathbf{{p}}}}}_{k} &= - \left ( \frac{2}{\Delta t} {\mathbf{{p}}}_{k} + \dot{\mathbf{{p}}}_{k} \right ) \end{aligned} \, \right \} . $$

By introducing Eq. (21) into Eq. (20), the dynamic equilibrium for the coupled system, at time-step $\left ( k+1 \right )$, can be written as:

23 $$ \left . \begin{aligned} {\mathbf{{M}}} {\mathbf{{z}}}_{k+1} + {\frac{\Delta t^{2}}{4}} {\boldsymbol{\Phi }}_{{\mathbf{{z}}}_{k+1}}^{ \text{T}} \left ( \alpha {\boldsymbol{\Phi }}_{k+1} + {\boldsymbol{\uplambda }}_{k+1} \right ) - {\frac{\Delta t^{2}}{4}} {\mathbf{{Q}}}_{k+1} + { \frac{\Delta t^{2}}{4}} {\mathbf{{M}}} \check{\ddot{\mathbf{{z}}}}_{k} = 0 \\ {\frac{\Delta t}{2}} {\mathbf{{p}}}_{k+1} - {\frac{\Delta t^{2}}{4}} {\mathbf{{v}}}_{k+1} + {\frac{\Delta t^{2}}{4}} \check{\dot{\mathbf{{p}}}}_{k} = 0 \end{aligned} \, \right \} $$

Equation (23) is a nonlinear system of equations that can be denoted as ${\acute{\mathbf{{f}}}} \left ( {\bar{\mathbf{{x}}}}_{k+1} \right ) = \mathbf{{0}}$, where ${\bar{\mathbf{{x}}}} = \left [ {\mathbf{{z}}}^{\text{T}}, {\mathbf{{p}}}^{\text{T}} \right ]^{\text{T}}$. Such nonlinear system of equations can be iteratively solved using the Newton–Raphson method with numerical derivatives. Here, the numerical derivatives are performed using the forward differentiation rule as in [17]. After every integration step, ${\dot{\mathbf{{z}}}}$ and ${\ddot{\mathbf{{z}}}}$ are corrected using the mass-damping-stiffness-orthogonal projections [3, 8], as shown in Eqs. (10) and (11).

## Design of state estimator

In this study, the state estimator is obtained by combining a coupled multibody system, described in Sect. 2.3, with the indirect Kalman filter [13, 33]. Here, the coupled multibody system is used without any modifications in its formulation. Thus, any multibody and hydraulic formulations or integrators can be used, including implicit integrators, projections and dependent coordinates, as is the case in this study. The eEKF-EJ method, described in [31], is extended to incorporate the pressure terms of a hydraulically actuated system.

**Fig. 3:**
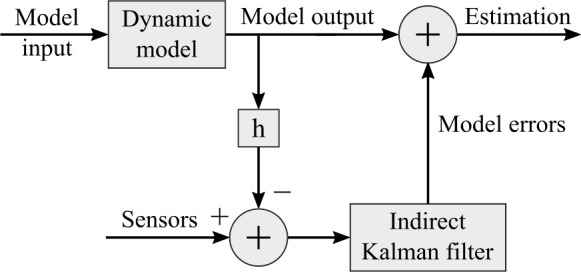
Schematic flow diagram of the indirect Kalman filter in each time-step

In this indirect Kalman filter, the filter estimates the error in the coupled multibody system, explained in Sect. 2.3, at each time-step of the simulation and the error is corrected with measurements of the real system. Figure 3 illustrates a simplified scheme of this formulation.

For each time-step of this filter, one step of the coupled multibody system is simulated to obtain its relative joint coordinates, ${\mathbf{{z}}}$, relative joint velocities, ${\dot{\mathbf{{z}}}}$, and pressures, ${\mathbf{{p}}}$. The state vector for the coupled multibody system can be written as ${\mathbf{{x}}} = \left [ \left ( \Delta {\mathbf{{z}}}^{\text{i}}\right )^{ \text{T}}, \left ( \Delta {\dot{\mathbf{{z}}}}^{\text{i}}\right )^{\text{T}}, \Delta {\mathbf{{p}}}^{\text{T}} \right ]^{\text{T}}$. Here, ${\Delta {\mathbf{{z}}}^{\text{i}}}$ and ${\Delta {\dot{\mathbf{{z}}}}^{\text{i}}}$ are respectively the errors in the relative joint coordinates and velocities of the degrees of freedom of the multibody system and $\Delta {\mathbf{{p}}}$ is the error in the pressures of the hydraulic subsystem. Next, the state vector, ${\hat{\mathbf{{x}}}}$, is estimated such that:

24 $$ \left . \begin{aligned} {\hat{\mathbf{{z}}}^{\text{i}}} &= {\mathbf{{z}}}^{\text{i}} + \Delta {\mathbf{{z}}}^{ \text{i}} \\ {\hat{\dot{\mathbf{{z}}}}^{\text{i}}} &= {\dot{\mathbf{{z}}}}^{\text{i}} + \Delta { \dot{\mathbf{{z}}}}^{\text{i}} \\ {\hat{\mathbf{{p}}}} &= {\mathbf{{p}}} + \Delta {\mathbf{{p}}} \end{aligned} \, \right \} $$

where ${\mathbf{{z}}}^{\text{i}}$ and ${\dot{\mathbf{{z}}}}^{\text{i}}$ are, respectively, the values of the relative joint coordinates and velocities of the degrees of freedom of the mechanism predicted by the coupled multibody system prior to the correction phase; ${\hat{\mathbf{{z}}}^{\text{i}}}$ and ${\hat{\dot{\mathbf{{z}}}}^{\text{i}}}$ are respectively their estimated values after the correction phase; and ${\mathbf{{p}}}$ is the value of the pressures predicted by the coupled multibody system prior to the correction phase and ${\hat{\mathbf{{p}}}}$ is its estimated value after the correction phase. It is assumed that a linearized estimator can be well suited to a problem of estimating small displacements in a linearized neighborhood or tangent space of the nonlinear manifold of the state space of the mechanism.

The propagation phase or the prediction stage can be performed as follows:

25 $$ {\hat{\mathbf{{x}}}}^{-}_{k} = {\mathbf{{0}}} $$

26 $$ {\mathbf{{P}}}^{-}_{k} = \left ( {\mathbf{{f}}}_{\mathbf{{x}}} \right )_{k-1} {\mathbf{{P}}}^{+}_{k-1} \left ( {\mathbf{{f}}}_{\mathbf{{x}}} \right )_{k-1}^{\text{T}} + { \boldsymbol{\textstyle {\sum }}}{}^{P}_{k-1} $$

where ${\hat{\mathbf{{x}}}}^{-}_{k}$ is the predicted mean of the state, also knows as ‘a priori’ state estimation, and ${\mathbf{{P}}}^{-}_{k}$ is its associated covariance matrix, ${\mathbf{{P}}}^{+}_{k-1}$ is the covariance matrix associated with the corrected mean of the state, ${\hat{\mathbf{{x}}}}^{+}_{k-1}$, from the previous time-step, ${\mathbf{{f}}}_{\mathbf{{x}}}$ is the Jacobian matrix of the discrete transition model, ${\mathbf{{f}}} \left ( \cdot \right )$, of the system with respect to the estimated state ${\hat{\mathbf{{x}}}}$, and $\boldsymbol{\textstyle {\sum }}{}^{P}_{k-1}$ is the covariance matrix of the plant noise, which stands for the additional uncertainty of the new state. The value of $\boldsymbol{\textstyle {\sum }}{}^{P}_{k-1}$ is physically attributed to the incorrect forces and errors in modeling the mechanism, such as inertia values, initial conditions, and many more. Note that Eqs. (25) and (26) are the equations obtained when one applies the conventional Kalman filter to the tracking error of a model [13, 33]. The matrix ${\mathbf{{f}}}_{\mathbf{{x}}}$ in Eq. (26), which follows a forward Euler integration, can be written as:

27 $$ \begin{aligned} & {\mathbf{{f}}}_{\mathbf{{x}}} \equiv \frac{\partial {\mathbf{{f}}}}{\partial {\hat{\mathbf{{x}}}}} = \frac{\partial }{\partial \{ {\hat{\mathbf{{z}}}^{\text{i}}}, {\hat{\dot{\mathbf{{z}}}}^{\text{i}}}, {\hat{\mathbf{{p}}}} \}} \begin{bmatrix} {\hat{\mathbf{{z}}}^{\text{i}}} + {\Delta t} {\hat{\dot{\mathbf{{z}}}}^{\text{i}}} + \frac{1}{2}{\Delta t^{2}} {\ddot{\mathbf{{z}}}}^{\text{i}} \\ {\hat{\dot{\mathbf{{z}}}}^{\text{i}}} + {\Delta t} {\ddot{\mathbf{{z}}}}^{\text{i}} \\ {\hat{\mathbf{{p}}}} + {\Delta t} {\dot{\mathbf{{p}}}} \end{bmatrix} \\ & \simeq \begin{bmatrix} \left ( {\mathbf{{I}}}_{f}+\frac{1}{2} \frac{\partial \Delta {\ddot{\mathbf{{z}}}}^{\text{i}}}{\partial {\mathbf{{z}}}^{\text{i}}}{ \Delta t^{2}} \right ) & \left ( {\mathbf{{I}}}_{f}{\Delta t}+\frac{1}{2} \frac{\partial \Delta {\ddot{\mathbf{{z}}}}^{\text{i}}}{\partial {\dot{\mathbf{{z}}}}^{\text{i}}}{ \Delta t^{2}} \right ) & \left ( \frac{1}{2} \frac{\partial \Delta {\ddot{\mathbf{{z}}}}^{\text{i}}}{\partial {\mathbf{{p}}}}{ \Delta t^{2}} \right ) \\ \left ( \frac{\partial \Delta {\ddot{\mathbf{{z}}}}^{\text{i}}}{\partial {\mathbf{{z}}}^{\text{i}}}{ \Delta t} \right ) & \left ( {\mathbf{{I}}}_{f}+ \frac{\partial \Delta {\ddot{\mathbf{{z}}}}^{\text{i}}}{\partial {\dot{\mathbf{{z}}}}^{\text{i}}}{ \Delta t} \right ) & \left ( \frac{\partial \Delta {\ddot{\mathbf{{z}}}}^{\text{i}}}{\partial {\mathbf{{p}}}}{ \Delta t} \right ) \\ \left ( {\mathbf{{0}}}_{n_{p} \times f} \right ) & \left ( {\mathbf{{0}}}_{n_{p} \times f} \right ) & \left ( {\mathbf{{I}}}_{n_{p}} \right ) \end{bmatrix} \end{aligned} $$

where ${\mathbf{{I}}}_{f}$ is a ${\left (f \times f\right )}$ identity matrix in which $f$ is the number of degrees of freedom of the multibody system, ${\mathbf{{I}}}_{n_{p}}$ is a ${\left ({n_{p}} \times {n_{p}}\right )}$ identity matrix in which ${n_{p}}$ is the number of pressures modeled in the hydraulic subsystem, ${\Delta t}$ is the time-step, and ${\mathbf{{0}}}_{n_{p} \times f}$ is a ${\left (n_{p} \times f\right )}$ zero matrix. In Eq. (27), the blocks at positions $\left (3, 1\right )$ and $\left (3, 2\right )$ are set to ${\mathbf{{0}}}$ because the evolution of pressure with respect to position and velocity is unknown. For simplicity, it is assumed that $\frac{\partial \Delta {\dot{\mathbf{{p}}}}}{\partial {\mathbf{{p}}}} = {\mathbf{{0}}}$. In the case of complex force models, the partial derivatives in Eq. (27) are difficult to calculate [31], and for this reason they are obtained numerically in this study using the forward differentiation rule [17]. As this method operates in the transformed state-space of errors, the predicted mean of the state, ${\hat{\mathbf{{x}}}}^{-}_{k}$, is always zero, as shown in Eq. (25). In other words, the filter initially assumes that the dynamic model made a perfect work in tracking the real system.

The correction phase or the state-update stage is similar to the ones found in a conventional DEKF and can be written as follows [32]:

28 $$ {\mathbf{{y}}}_{k} = {\mathbf{{o}}}_{k} - {\mathbf{{h}}} \left ( {\mathbf{{z}}}_{k}, { \dot{\mathbf{{z}}}}_{k}, {\mathbf{{p}}}_{k} \right ) $$

29 $$ {\mathbf{{S}}}_{k} = \left ( {\mathbf{{h}}}_{\mathbf{{x}}} \right )_{k} {\mathbf{{P}}}^{-}_{k} \left ( {\mathbf{{h}}}_{\mathbf{{x}}} \right )_{k}^{\text{T}} + { \boldsymbol{\textstyle {\sum }}} {}^{S}_{k} $$

30 $$ {\mathbf{{K}}}_{k} = {\mathbf{{P}}}^{-}_{k} \left ( {\mathbf{{h}}}_{\mathbf{{x}}} \right )_{k}^{ \text{T}} {\mathbf{{S}}}_{k}^{-1} $$

31 $$ {\hat{\mathbf{{x}}}}^{+}_{k} = {\mathbf{{0}}} + {\mathbf{{K}}}_{k} {\mathbf{{y}}}_{k} $$

32 $$ {\mathbf{{P}}}^{+}_{k} = \left [ {\mathbf{{I}}}_{\left ( 2f + {n_{p}} \right )} - { \mathbf{{K}}}_{k} \left ( {\mathbf{{h}}}_{\mathbf{{x}}} \right )_{k} \right ] {\mathbf{{P}}}^{-}_{k} $$

where ${\mathbf{{y}}}_{k}$ is the error or innovation between virtual measurements ${\mathbf{{h}}} \left ( \cdot \right )$ and their actual measurements ${\mathbf{{o}}}_{k}$. Note that the virtual measurements ${\mathbf{{h}}} \left ( {\mathbf{{z}}}_{k}, {\dot{\mathbf{{z}}}}_{k}, {\mathbf{{p}}}_{k} \right )$ are built using the coordinates and pressures of the coupled multibody system instead of the states of the filter because the states of the filter are the errors, and the errors are always set to zero until after correction [31]. In Eq. (29), ${\mathbf{{S}}}_{k}$ is the covariance matrix of the innovation that represents the uncertainty of the system state projected by the sensor function $\left [ \left ( {\mathbf{{h}}}_{\mathbf{{x}}} \right )_{k} {\mathbf{{P}}}^{-}_{k} \left ( {\mathbf{{h}}}_{\mathbf{{x}}} \right )_{k}^{\text{T}} \right ]$ and an additional sensor Gaussian noise, $\boldsymbol{\textstyle {\sum }}{}^{S}_{k}$, known as the covariance matrix of the measurement noise. Small values of ${\mathbf{{S}}}_{k}$ imply that actual measurements or observations introduce valuable information that constrains the estimation of the system state. Here, ${\mathbf{{h}}}_{\mathbf{{x}}}$ is the Jacobian matrix of the measurement model, ${\mathbf{{h}}} \left ( \cdot \right )$, with respect to the state ${\mathbf{{x}}}$. Note that the expression of ${\mathbf{{h}}}_{\mathbf{{x}}}$ is obtained similarly to an equivalent conventional Kalman filter because the partial derivatives with respect to the errors in the states have the same value as the partial derivatives with respect to the states. In Eq. (30), ${\mathbf{{K}}}_{k}$ is the Kalman gain, which is a temporary term, to update or correct the predicted mean of the state and its covariance. In Eqs. (31) and (32), ${\hat{\mathbf{{x}}}}^{+}_{k}$ is the corrected mean of the state, also known as ‘a posteriori’ state estimation, and ${\mathbf{{P}}}^{+}_{k}$ is its associated covariance matrix. The covariance matrix ${\mathbf{{P}}}^{+}_{k}$ is used as input for the next iteration of this iterative filter at the next time-step.

After the correction phase, the estimated errors, $\Delta {\hat{\mathbf{{z}}}^{\text{i}}}$, $\Delta {\hat{\dot{\mathbf{{z}}}}^{\text{i}}}$ and $\Delta {\hat{\mathbf{{p}}}}$ are used in Eq. (24) to solve for ${\hat{\mathbf{{z}}}^{\text{i}}}$, ${\hat{\dot{\mathbf{{z}}}}^{\text{i}}}$, and ${\hat{\mathbf{{p}}}}$. As the corrections are expected to be small, a linearization of the position problem can be solved using ${\boldsymbol{\Phi }}_{\mathbf{{z}}} \Delta {\mathbf{{z}}} = {\mathbf{{0}}}$. As a consequence, the estimated error of the dependent relative joint coordinates, $\Delta {\hat{\mathbf{{z}}}^{\text{d}}}$, of the multibody system can be written in terms of $\Delta {\hat{\mathbf{{z}}}^{\text{i}}}$ as:

33 $$ \Delta {\hat{\mathbf{{z}}}^{\text{d}}} = - \left ( {\boldsymbol{\Phi }}_{\mathbf{{z}}}^{ \text{d}} \right )^{-1} {\boldsymbol{\Phi }}_{\mathbf{{z}}}^{\text{i}} \Delta { \hat{\mathbf{{z}}}^{\text{i}}} $$

where ${\boldsymbol{\Phi }}_{\mathbf{{z}}}^{\text{d}}$ and ${\boldsymbol{\Phi }}_{\mathbf{{z}}}^{\text{i}}$ are, respectively, the dependent and independent columns of the Jacobian matrix ${\boldsymbol{\Phi }}_{\mathbf{{z}}}$. It is assumed that ${\boldsymbol{\Phi }}_{\mathbf{{z}}}^{\text{d}}$ is invertible. The complete set of estimated errors of the relative joint coordinates, $\Delta {\hat{\mathbf{{z}}}} = \left [ \left ( \Delta {\hat{\mathbf{{z}}}^{ \text{i}}} \right )^{\text{T}}, \left ( \Delta {\hat{\mathbf{{z}}}^{\text{d}}} \right )^{\text{T}} \right ]^{\text{T}}$, can be used to estimate the relative joint coordinates, ${\hat{\mathbf{{z}}}}$, as:

34 $$ {\hat{\mathbf{{z}}}} = {\mathbf{{z}}} + \Delta {\hat{\mathbf{{z}}}} $$

where ${\mathbf{{z}}}$ is the value of the relative joint coordinates predicted by the coupled multibody system prior to the correction phase. Note that this approach to solving the position problem is an approximation to avoid solving it iteratively, thus, a perfect fulfillment of the constraints at position level is not expected. The estimator corrects the states of the multibody model whenever measurements are available and this may lead to imperfect fulfillment of the constraints. However, the multibody formulation imposes the fulfillment of constraints at every time-step, and therefore the errors are usually acceptable for most applications [31]. Nevertheless, when the highest possible accuracy is required, then the exact position problem must be solved. Once the correction of the position estimation is applied, the correction of the velocity estimation is solved using the coordinate partitioning method as in [20]:

35 $$ {\hat{\dot{\mathbf{{z}}}}^{\text{d}}} = - \left ( {\boldsymbol{\Phi }}_{\mathbf{{z}}}^{ \text{d}} \right )^{-1} {\boldsymbol{\Phi }}_{\mathbf{{z}}}^{\text{i}} { \hat{\dot{\mathbf{{z}}}}^{\text{i}}} $$

where ${\hat{\dot{\mathbf{{z}}}}^{\text{d}}}$ are the estimated values of the dependent relative joint velocities such that ${\hat{\dot{\mathbf{{z}}}}} = \left [ \left ( {\hat{\dot{\mathbf{{z}}}}^{\text{i}}} \right )^{\text{T}}, \left ( {\hat{\dot{\mathbf{{z}}}}^{\text{d}}} \right )^{ \text{T}} \right ]^{\text{T}}$. Once the dynamic model is corrected using ${\hat{\mathbf{{z}}}}$, ${\hat{\dot{\mathbf{{z}}}}}$, and ${\hat{\mathbf{{p}}}}$, then the expected error becomes ${\hat{\mathbf{{x}}}}^{+}_{k} = {\mathbf{{0}}}$

## Covariance matrices of plant and measurement noises

In the application of the Kalman filter, the tuning of parameters, such as the covariance matrices of the plant and measurement noise, that is, $\boldsymbol{\textstyle {\sum }}{}^{P}$ and $\boldsymbol{\textstyle {\sum }}{}^{S}$, is crucial. If $\boldsymbol{\textstyle {\sum }}{}^{P}$ and $\boldsymbol{\textstyle {\sum }}{}^{S}$ are not properly defined, then a nonlinear system can become unstable even though all other parameters of the filter are suitably tuned.

### Structure of plant noise

In case of a multibody model only, the geometrical properties can be precisely modeled, whereas, the accurate modeling of the forces and the actual distribution of the masses are complex in practice. As a consequence, the system deviates from the ideal behavior and errors occur at the acceleration level. Furthermore, the integration process and the multibody formulation may cause additional errors, however, they are negligible compared to the previous error. Therefore, in this study, only the acceleration terms are included in the covariance matrix of the plant noise for the multibody system, as in [31, 32]. Furthermore, since the states considered in the state estimator do not contain acceleration terms, $\boldsymbol{\textstyle {\sum }}{}^{P}$ must be calculated from its continuous-time counterpart. For example, it can be computed using Van Loan’s method [36] of integration as in [32]. Note that if acceleration errors were considered in the proposed estimator, then the acceleration noise could be used in its discrete form as in [31]. The structure of $\boldsymbol{\textstyle {\sum }}{}^{P}$ for the multibody system with position and velocity estimation can be written as [32]:

36 $$\sum {}^{P}=\left[\begin{array}{cc} \sigma_{\ddot{z}}^{2}\frac{\Delta t^{3}}{3}I_{f} & \sigma_{\ddot{z}}^{2}\frac{\Delta t^{2}}{2}I_{f}\\ \sigma_{\ddot{z}}^{2}\frac{\Delta t^{2}}{2}I_{f} & \sigma_{\ddot{z}}^{2}\Delta tI_{f} \end{array}\right]$$

where $\sigma _{\ddot{\mathbf{{z}}}}$ is the variance of all components of the continuous plant noise at acceleration level.

In this study, a hydraulic subsystem is coupled with the multibody system, therefore the pressure level noise coming from the hydraulics can be directly incorporated in $\boldsymbol{\textstyle {\sum }}{}^{P}$ as:

37 $$\sum {}^{P}=\left[\begin{array}{ccc} \sigma_{\ddot{z}}^{2}\frac{\Delta t^{3}}{3}I_{f} & \sigma_{\ddot{z}}^{2}\frac{\Delta t^{2}}{2}I_{f} & 0_{f\times n_{p}}\\ \sigma_{\ddot{z}}^{2}\frac{\Delta t^{2}}{2}I_{f} & \sigma_{\ddot{z}}^{2}\Delta tI_{f} & 0_{f\times n_{p}}\\ 0_{n_{p}\times f} & 0_{n_{p}\times f} & \sigma_{p,D}^{2}I_{n_{p}} \end{array}\right]$$

where $\sigma _{{\mathbf{{p}}},D}$ is the variance of all components of the discrete plant noise at the pressure level. Note that both $\sigma _{\ddot{\mathbf{{z}}}}$ and $\sigma _{{\mathbf{{p}}},D}$ are tuned by trial and error. However, $\sigma _{\ddot{\mathbf{{z}}}}$ is independent of the simulation time-step, as it is a continuous variance, whereas, $\sigma _{{\mathbf{{p}}},D}$ should be modified proportionally to the simulation time-step as it is a discrete variance.

### Structure of measurement noise

In this section, the structure of the covariance matrix of the measurement noise is presented. It should be noted that the structure presented here is equally applicable to most real sensors currently in use. Nevertheless, the measurements in this study are built from a dynamic model of the coupled multibody system that has zero modeling error and acts as a real system, thus providing the ground truth. White Gaussian noise is generated and added to the measurements to represent the noise properties of real sensors. Therefore, the measurement noise properties are already known and are used to obtain the covariance matrix of the measurement noise. For example, the structure of $\boldsymbol{\textstyle {\sum }}{}^{S}$ with position and pressure sensors takes the form as:

38 $$ \boldsymbol{\textstyle {\sum }}{}^{S} = \begin{bmatrix} \left ({\sigma ^{\prime }_{\mathbf{{z}}}}\right )^{2} {\mathbf{{I}}}_{f} & {\mathbf{{0}}}_{f \times n_{p}} \\ {\mathbf{{0}}}_{n_{p} \times f} & \left ({\sigma ^{\prime }_{\mathbf{{p}}}}\right )^{2} { \mathbf{{I}}}_{n_{p}} \end{bmatrix} $$

where ${\sigma ^{\prime }_{\mathbf{{z}}}}$ and ${\sigma ^{\prime }_{\mathbf{{p}}}}$ are the standard deviations of the measurement noise at the position and pressure levels, respectively. Note that a similar sequence of pseudo-random noise is used in different combinations of sensors to ensure a fair comparison.

## The case study of a hydraulically actuated four-bar mechanism

**Fig. 4:**
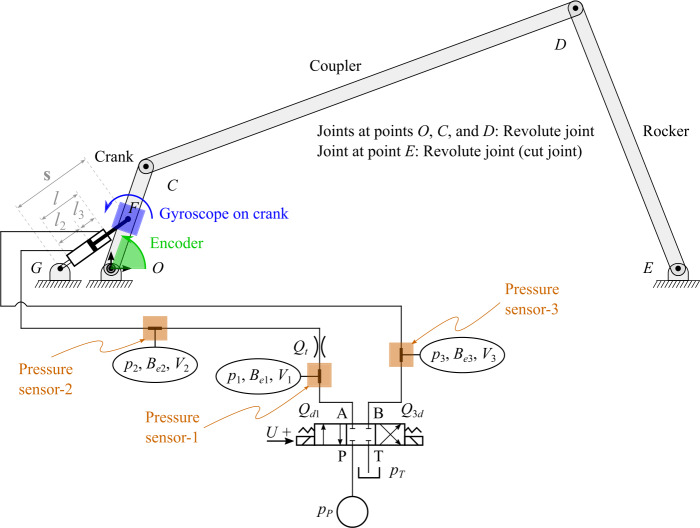
A four-bar mechanism actuated by a double-acting hydraulic cylinder

In this study, a hydraulically actuated four-bar mechanism, as shown in Fig. 4, is used to demonstrate the state estimator explained in Sect. 3. The four-bar mechanism is modeled using the semi-recursive formulation, explained in Sect. 2.1. It has three bodies: crank, coupler and rocker and four revolute joints, where the joint between the rocker and the ground is a cut-joint for which two loop-closure constraint equations are introduced. Note that although a planar mechanism is presented in this study, the implementation of the methods is carried out in the Matlab environment in three-dimensions. The system has one degree of freedom. Note that the crank, coupler, and rocker are assumed to be rectangular beams, whose lengths are $L_{1} = 2\text{ m}$, $L_{2} = 8\text{ m}$, and $L_{3} = 5\text{ m}$, and masses are $m_{1} = 2\text{ kg}$, $m_{2} = 8\text{ kg}$, and $m_{3} = 5\text{ kg}$, respectively. The moment of inertia of the beams is considered as $\frac{m{L}^{2}}{12}$, where $m$ is its mass and $L$ is its length. In the inertial reference frame, the positions of the bodies are represented by ${z_{1}}$, ${z_{2}}$ and ${z_{3}}$, respectively. The position vector of points ${E}$, ${F}$ and ${G}$ are, respectively, ${\mathbf{{r}}}_{E} = \left [ 10, 0, 0 \right ]^{\text{T}}\text{ m}$, ${\mathbf{{r}}}_{F} = \left [ \frac{L_{1}}{2} \cos {(z_{1})}, \frac{L_{1}}{2} \sin {(z_{1})}, 0 \right ]^{\text{T}}\text{ m}$ and ${\mathbf{{r}}}_{G} = \left [ - \frac{L_{1}}{2}, 0, 0 \right ]^{\text{T}}\text{ m}$, and point ${F}$ is located at the center of mass of the crank.

The actuation of the four-bar mechanism is carried out using the hydraulic circuit shown in Fig. 4. The hydraulic circuit consists of a pump with a constant pressure source ${p_{P}}$, a tank with a constant pressure ${p_{T}}$, a directional control valve with a control signal $U$, a throttle valve, a double-acting hydraulic cylinder and connecting hoses. It is assumed that the hydraulic circuit is ideal, that is, it has no leakage. According to the lumped fluid theory, the hydraulic circuit is divided into three control volumes, ${V_{1}}$, ${V_{2}}$, and ${V_{3}}$, whose respective pressures, ${p_{1}}$, ${p_{2}}$, and ${p_{3}}$, are computed by using Eq. (12) as:

39 $$ \left . \begin{aligned} {{\dot{p}}_{1}} &= \frac{B_{e1}}{V_{1}} \left ( {Q_{d1}} - {Q_{t}} \right ) \\ {{\dot{p}}_{2}} &= \frac{B_{e2}}{V_{2}} \left ( {Q_{t}} - {A_{2}} { \dot{s}} \right ) \\ {{\dot{p}}_{3}} &= \frac{B_{e3}}{V_{3}} \left ( {A_{3}} {\dot{s}} - {Q_{3d}} \right ) \end{aligned} \, \right \} $$

where ${B_{e1}}$, ${B_{e2}}$, and ${B_{e3}}$ are the effective bulk modulus of the respective sections calculated from Eq. (13), ${Q_{t}}$ is the volume flow rate calculated from Eq. (14), ${Q_{d1}}$ and ${Q_{3d}}$ are the volume flow rates calculated from Eq. (16), ${A_{2}}$ and ${A_{3}}$ are, respectively, the areas of the piston side and the piston-rod side within a cylinder, and ${\dot{s}}$ is the actuator velocity. In Eq. (39), ${V_{1}}$, ${V_{2}}$, and ${V_{3}}$ are calculated as:

40 $$ \left . \begin{aligned} {V_{1}} &= {V_{h_{1}}} \\ {V_{2}} &= {V_{h_{2}}} + {A_{2}} {l_{2}} \\ {V_{3}} &= {V_{h_{3}}} + {A_{3}} {l_{3}} \end{aligned} \, \right \} $$

where ${V_{h_{1}}}$, ${V_{h_{2}}}$, and ${V_{h_{3}}}$ are the volumes of the respective hoses, and ${l_{2}}$ and ${l_{3}}$ are, respectively, the length of the piston side and the piston-rod side, that are calculated as:

41 $$ \left . \begin{aligned} {l_{2}} &= l_{2_{0}} + {s_{0}} - {\mid {\mathbf{{s}}} \mid } \\ {l_{3}} &= l_{3_{0}} - {s_{0}} + {\mid {\mathbf{{s}}} \mid } \end{aligned} \, \right \} $$

where $l_{2_{0}}$ and $l_{3_{0}}$ are, respectively, the values of ${l_{2}}$ and ${l_{3}}$ at $t=0$, ${\mid {\mathbf{{s}}} \mid }$ is the actuator length of the hydraulic cylinder, and $s_{0}$ is its value at $t=0$. Note that the length of the hydraulic cylinder is ${l} = {l_{2}} + {l_{3}}$. The values of ${\dot{s}}$ in Eq. (39) and ${\mid {\mathbf{{s}}} \mid }$ in Eq. (41) are computed by using ${z_{1}}$ and ${{\dot{z}}_{1}}$ as:

42 $$ \left . \begin{aligned} {\mathbf{{s}}} &= {\mathbf{{r}}}_{F} - {\mathbf{{r}}}_{G} \\ {\dot{s}} &= \frac{d{\mid {\mathbf{{s}}} \mid }}{dt} = {\dot{{\mathbf{{s}}}}} \cdot \frac{{\mathbf{{s}}}}{{\mid {\mathbf{{s}}} \mid }} = {\dot{\mathbf{{r}}}}_{F} \cdot \frac{{\mathbf{{s}}}}{{\mid {\mathbf{{s}}} \mid }} \end{aligned} \, \right \} $$

where ${\dot{\mathbf{{r}}}}_{F}$ is the velocity vector of point ${F}$. For simplicity, the force ${F_{c}}$ obtained from Eq. (19) is expressed in the form of Eq. (7) as: ${\mathbf{{F}}}_{c} = \left [ \frac{s_{\text{X}}}{{\mid {\mathbf{{s}}} \mid }}{F_{c}}, \frac{s_{\text{Y}}}{{\mid {\mathbf{{s}}} \mid }}{F_{c}}, \frac{s_{\text{Z}}}{{\mid {\mathbf{{s}}} \mid }}{F_{c}} \right ]^{\text{T}}$, where ${s_{\text{X}}}$, ${s_{\text{Y}}}$ and ${s_{\text{Z}}}$ are the components of vector ${\mathbf{{s}}}$ along the axes of the inertial reference frame. The value of ${F_{c}}$ at $t=0$ can be computed from the static configuration as: ${F_{c_{0}}} = \left ( \frac{{m_{1}} g \cos {(z_{1_{0}})}}{\sin {\left (\frac{z_{1_{0}}}{2}\right )}} + \frac{(m_{2} g + m_{3} g) \cos (z_{1_{0}} + z_{2_{0}} + z_{3_{0}}) \sin {(z_{2_{0}})}}{{\sin {\left (\frac{z_{1_{0}}}{2} \right )}} \sin {(2 z_{1_{0}} + 2 z_{2_{0}} + z_{3_{0}})}} \right )$, where $z_{1_{0}}$, $z_{2_{0}}$, and $z_{3_{0}}$ are the values of $z_{1}$, $z_{2}$, and $z_{3}$ at $t=0$. In the static configuration, $p_{1_{0}} = p_{2_{0}}$, and from Eq. (19), ${p_{2_{0}}} = \left ({F_{c_{0}}} + {p_{3_{0}}}{A_{3}} \right ) / {A_{2}}$, where $p_{1_{0}}$, $p_{2_{0}}$, and $p_{3_{0}}$ are the respective values of $p_{1}$, $p_{2}$, and $p_{3}$ at $t=0$. Note that the friction is neglected in static configuration. The four-bar structure is hydraulically actuated for 6 s, such that:

43 $$ U_{ref} = \textstyle\begin{cases} 0 & t < 1 \hspace{0.1cm} \text{s}, \hspace{0.25cm} 2.5 \hspace{0.1cm} \text{s} \leq t < 3.5 \hspace{0.1cm} \text{s}, \hspace{0.25cm} t \leq 6 \hspace{0.1cm} \text{s} \\ 10 & 1 \hspace{0.1cm} \text{s} \leq t < 2.5 \hspace{0.1cm} \text{s} \\ -10 & 3.5 \hspace{0.1cm} \text{s} \leq t < 5 \hspace{0.1cm} \text{s} \end{cases} $$

where ${t}$ is the simulation run time. The set of variables used inside the trapezoidal integration scheme to solve the coupled multibody system can be written as ${\bar{\mathbf{{x}}}} = \left [ {\mathbf{{z}}}^{\text{T}},\; {\mathbf{{p}}}^{\text{T}} \right ]^{\text{T}} = \left [ {z_{1}},\; {z_{2}},\; {z_{3}},\; {p_{1}}, \; {p_{2}},\; {p_{3}} \right ]^{\text{T}}$. In the trapezoidal integration scheme, the error tolerance for position is $1 \times 10^{-10}\text{ rad}$ and for pressure is $1 \times 10^{-2}\text{ Pa}$. The voltage, which corresponds to the spool position is integrated using the trapezoidal method and its error tolerance is $1 \times 10^{-10}$ V. The penalty factor, $\alpha $, used in the study (Eq. (8)) is $1 \times 10^{9}$. The parameters of the hydraulic circuit are shown in Table 1.

**Table 1:** Parameters of the hydraulic circuit

Parameter	Symbol	Value
Pressure of the tank (atmospheric pressure)	${p_{T}}$	0.1 MPa
Semi-empirical flow rate constant of the directional control valve	${C_{v_{d}}}$	2.138 ×10^−8^ m^3^/s $\sqrt{\text{ Pa}}$
Volume of the hose (section-1)	${V_{h_{1}}}$	4.71 ×10^−5^ m^3^
Volume of the hose (section-2)	${V_{h_{2}}}$	3.14 ×10^−5^ m^3^
Volume of the hose (section-3)	${V_{h_{3}}}$	7.85 ×10^−5^ m^3^
Area of the throttle valve	${A_{t}}$	2.83 ×10^−5^ m^2^
Flow discharge coefficient of the throttle valve	${C_{d}}$	0.8
Density of the oil	*ρ*	850 kg/m^3^
Bulk modulus of the hoses	${B_{h}}$	550 MPa
Bulk modulus of the oil	${B_{oil}}$	1500 MPa
Bulk modulus of the hydraulic cylinder	${B_{c}}$	31500 MPa
Diameter of the piston	${d_{2}}$	80 mm
Diameter of the piston-rod	${d_{3}}$	35 mm
Length of the cylinder/piston	*l*	0.9 m
Initial actuator length	${s_{0}}$	1.73 m

In this study, dynamic models are used to provide a fair comparison for the implemented state estimator. The first reference model represents the actual mechanism that is modeled without any modeling error and is referred to as the “real system”, as in [31, 32], thus, providing the ground truth. The “real system” in this study can also be referred to as the “ground truth” or “reference model”. Measurements are obtained from this model with an addition of white Gaussian noise to represent the noise properties of the actual sensors. The second model represents an imperfect representation of the “real system” or “reference model”, with some parameters modified to simulate modeling errors, and is referred to as the “simulation model”. The “simulation model” in this study can also be referred to as the “model”, as in [31, 32], or the “imperfect model”. The modeling errors introduced in the simulation model can be seen in Table 2. Furthermore, the implementation of the indirect Kalman filter on the simulation model described above is referred to as the “state estimator”. The “state estimator” combines the “imperfect model” with noisy measurements obtained from the “reference model” to achieve the best possible estimations of the true state of the “reference model”, which is unknown. The “state estimator” in this study can also be referred to as the “state observer”. In the state estimator, the simulation model is corrected using the measurements of the real system, described above.

**Table 2:** Parametric difference between the real system and the simulation model

Parameter	Symbol	Real system	Simulation model
Gravity	*g*	-9.81 m/s^2^	-8.81 m/s^2^
Crank angle at *t* = 0	$z_{1_{0}}$	60^o^	71^o^
Pressure of the pump	${p_{P}}$	7.6 MPa	5.6 MPa
Pressure of $p_{3}$ at *t* = 0	$p_{3_{0}}$	3.5 MPa	3.0 MPa

The errors introduced in the simulation model compared to the real system are in the force model. Note that when modeling a coupled multibody system, the geometry and mass can be accurately defined. However, the force models might have uncertainties in the modeling [31]. Therefore, incorrect values for gravity, as in [31], and pump pressures, are considered in the simulation model compared to the real system, as shown in Table 2. This introduces an incorrect force model such that the dynamics of the “simulation model” or “imperfect model” are affected throughout the simulation. Consequently, the “simulation model” will be out of synchronization compared to the “real system” or “reference model”, just like any unmodeled force would affect. Furthermore, the ability to correct for the initial position and pressure errors in the simulation model provides a non-formal demonstration of the system observability.

For the present case study, the state vector considered for the state estimator is ${\mathbf{{x}}} = \left [ \left ( \Delta {\mathbf{{z}}}^{\text{i}}\right )^{ \text{T}}, \left ( \Delta {\dot{\mathbf{{z}}}}^{\text{i}}\right )^{\text{T}}, \Delta {\mathbf{{p}}}^{\text{T}} \right ]^{\text{T}} = \left [ \Delta {z_{1}}, \; \Delta {\dot{z}_{1}},\; \Delta {p_{1}},\; \Delta {p_{2}},\; \Delta {p_{3}} \right ]^{\text{T}}$. That is, ${z_{1}}$ is selected as the independent relative joint coordinate and assumed to be valid throughout the simulation, as is the case with a hydraulically actuated machinery. The initial values of ${z_{2}}$ and ${z_{3}}$ are derived from the initial value of ${z_{1}}$ shown in Table 2. To maintain the stability of the simulation process, the simulations are executed from static equilibrium. Note that gravity acts in the negative Y-direction. The initial covariance, ${\mathbf{{P}}}_{0}$, of the state estimator is a diagonal matrix whose first two diagonal elements are, respectively, $0.76 \times 10^{-2}\text{ rad}^{2}$ and $0.76 \times 10^{-2}\text{ rad}^{2}$/s^4^, and the last three elements are $22.5 \times 10^{7}\text{ Pa}^{2}$. In this study, the standard deviation of the measurement noise at the position, velocity and pressure levels are ${\sigma ^{\prime }_{\mathbf{{z}}}} = 1.745 \times 10^{-2}\text{ rad}$, $\sigma ^{\prime }_{\dot{\mathbf{{z}}}} = 9.839 \times 10^{-4}\text{ rad}/\text{s}$, and ${\sigma ^{\prime }_{\mathbf{{p}}}} = 0.15 \times 10^{5}\text{ Pa}$, respectively. Whereas, the values of the plant noise are $\sigma ^{2}_{\ddot{\mathbf{{z}}}} = 11.163 \times 10^{-2}\text{ rad}^{2}$/s^4^ and $\sigma ^{2}_{{\mathbf{{p}}},D} = 259.81 \times 10^{7}\text{ Pa}^{2}$, which are obtained by trial and error [31]. In this study, the simulations are run with a time-step of 1 ms, providing information about the coupled system at 1000 Hz.

In this study, a combination of position, velocity and pressure sensors is used, as shown in Fig. 4. The advantage of using a coupled multibody system inside a Kalman filter is that it provides a mean to obtain the Jacobian matrix of the measurement model, ${\mathbf{{h}}}_{\mathbf{{x}}}$, in a systematic way. All coordinates, velocities and pressures are available from the coupled multibody system, so that building the model of the sensors and obtaining their Jacobian is quite straightforward, as shown in the following subsections.

### Position sensor

The devices used to measure the angular position of a body are encoders, which are commonly used in all kinds of machines to monitor angular magnitudes. Thus, in this study, an encoder is used as a position sensor at the location of the crank of the four-bar mechanism, as shown in Fig. 4. It measures the angle of the crank, that is, ${z_{1}}$, such that the measurement model is ${\mathbf{{h}}} \left ( {\mathbf{{x}}} \right ) = \left [ {z_{1}} \right ]$. As the states considered in the state estimator are ${\mathbf{{x}}} = \left [ \Delta {z_{1}},\; \Delta {\dot{z}_{1}},\; \Delta {p_{1}}, \; \Delta {p_{2}},\; \Delta {p_{3}} \right ]^{\text{T}}$, thus, ${\mathbf{{h}}}_{\mathbf{{x}}}$ can be written as:

44 $$ {\mathbf{{h}}}_{\mathbf{{x}}} = \begin{bmatrix} 1,\; & 0,\; & 0,\; & 0,\; & 0 \end{bmatrix} $$

### Velocity sensor

The state estimator developed in this study is also tested by incorporating a velocity sensor. The most common devices used to measure the angular velocity of a body are micro-electro-mechanical systems (MEMS) gyroscopes. MEMS gyroscopes are used in many applications such as cell phones, robots and autonomous vehicles. For the presented case study, a gyroscope is installed on the crank, as shown in Fig. 4. Thus, it will measure the angular velocity of the crank, that is, ${\omega }_{1} = {\dot{z}_{1}}$, such that the measurement model is ${\mathbf{{h}}} \left ( {\mathbf{{x}}} \right ) = \left [ {\dot{z}_{1}} \right ]$ and ${\mathbf{{h}}}_{\mathbf{{x}}}$ can be written as:

45 $$ {\mathbf{{h}}}_{\mathbf{{x}}} = \begin{bmatrix} 0,\; & 1,\; & 0,\; & 0,\; & 0 \end{bmatrix} $$

### Pressure sensors

The pressures in a hydraulic circuit can be measured using pressure sensors. There are many pressure sensors that can be used, such as gauge pressure sensors, which measure pressure relative to atmospheric pressure. In this study, pressure sensors can be installed at three hydraulic control volumes in the hydraulic circuit, as shown in Fig. 4. When the pressure sensors are installed at control volumes $V_{1}$ and $V_{3}$, for example, then they measure pressures ${p_{1}}$ and ${p_{3}}$, such that the measurement model is ${\mathbf{{h}}} \left ( {\mathbf{{x}}} \right ) = \left [ {p_{1}},\; {p_{3}} \right ]^{\text{T}}$, and ${\mathbf{{h}}}_{\mathbf{{x}}}$ can be written as:

46 $$ {\mathbf{{h}}}_{\mathbf{{x}}} = \begin{bmatrix} 0,\; & 0,\; & 1,\; & 0,\; & 0 \\ 0,\; & 0,\; & 0,\; & 0,\; & 1 \end{bmatrix} $$

## Results and discussion

**Fig. 5:**
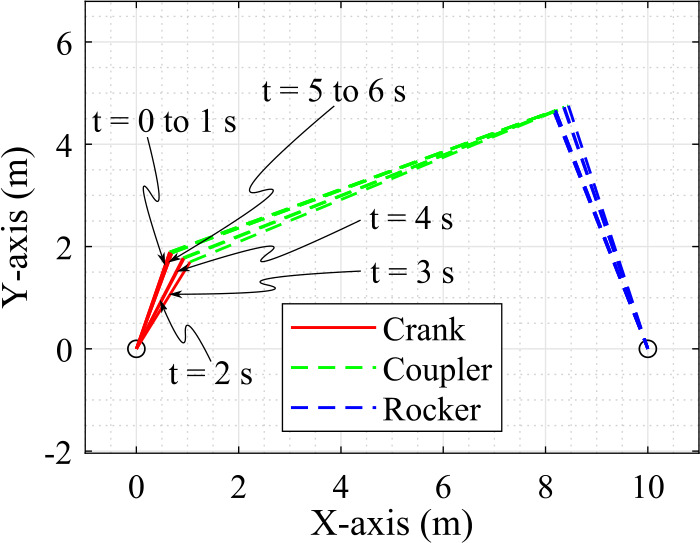
The position of bodies of the simulation model at every second with a time-step of 1 ms

This section presents the results of the state estimator applied to the hydraulically actuated four-bar mechanism, presented in Sect. 5. The position of the bodies of the simulation model at different instants of time is shown in Fig. 5. In this study, a non-formal demonstration of the system observability is provided by the initial position error of the crank angle and the initial pressure errors of the hydraulic control volumes. The system is observable only if the position error (i.e. the initial $11^{\text{o}}$ error of the crank angle) and the pressure errors (i.e. the initial 0.5 MPa error of pressure ${p_{3}}$, and 0.41 MPa error of pressures ${p_{1}}$ and ${p_{2}}$, each) are corrected with a set of sensors. Accordingly, four sets of sensor combinations are used, as shown in Table 3.

**Table 3:** Sensor combinations used in this study

	Position sensor	Velocity sensor	Pressure sensors
Sensor set-1	Crank	–	$p_{1}$, $p_{2}$, and $p_{3}$
Sensor set-2	Crank	Crank	$p_{1}$, $p_{2}$, and $p_{3}$
Sensor set-3	Crank	Crank	$p_{1}$ and $p_{2}$
Sensor set-4	Crank	Crank	$p_{1}$ and $p_{3}$

In this study, the simulations are run with a time-step of 1 ms, providing information about the coupled system at 1000 Hz. Furthermore, the sampling rates considered for the sensors are 1000 Hz (one measurement per simulation time-step); 500 Hz (one measurement available every two simulation time-steps), 200 Hz (one measurement every five simulation time-steps), and 100 Hz (one measurement every 10 simulation time-steps). The states of the estimator are not corrected when no measurements are available. Note that the simulation model contains high modeling errors, as shown in Table 2. It should be noted that this study focuses only on the estimation accuracy and not on the computational efficiency of the estimator. The computational efficiency is ignored in this study because numerical methods (for Eqs. (23) and (27)) are employed in the Matlab environment and thus computational efficiency is expected to be low.

### Testing with different sampling rate of sensors

**Fig. 6:**
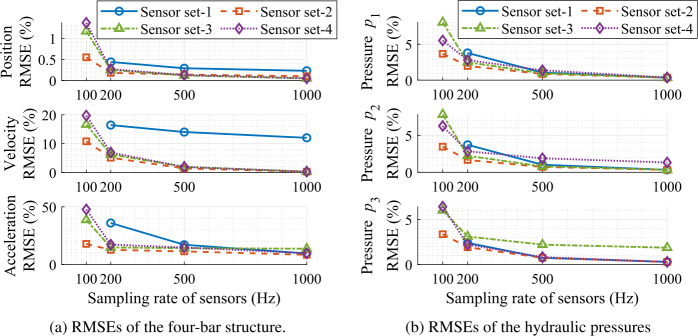
Root mean square errors of the state estimator, with respect to the absolute maximum value of the real systems, at different sampling rates of the sensors

Figure 6a presents the root mean square error (RMSE) of the different tests on the position level (the crank angle error), the velocity level (the crank angular rate error) and the acceleration level (the crank angular acceleration error). The RMSEs of the hydraulic pressures are provided in Fig. 6b. Note that the RMSEs are measured in $\%$ with respect to the absolute maximum value of the real system. It can be observed that sensor set-2 provides relatively better estimation accuracy compared to the other sensor sets because it utilizes all sensors at position, velocity and pressure levels. Furthermore, the accuracy of the estimation degrades when the sampling rate of the sensors is reduced.

Note that sensor set-1 is unable to provide a result at a sampling rate of 100 Hz. This is because the terms of the covariance matrix grow, resulting in higher corrections, which is more difficult for the multibody model to handle, thus, reaching a maximum number of iterations. To stabilize the multibody simulation at 100 Hz sampling rate with sensor combination-1, the values of the covariance matrix of the plant noise can be reduced depending on the application. Even though the plant covariance noise may not be optimal, it can help to have smaller corrections by compromising on accuracy that will not unstabilize the multibody simulation. However, this was not done in this study so that different sensor combinations can be tested under the same conditions.

### Accuracy of the work cycle estimation

**Fig. 7:**
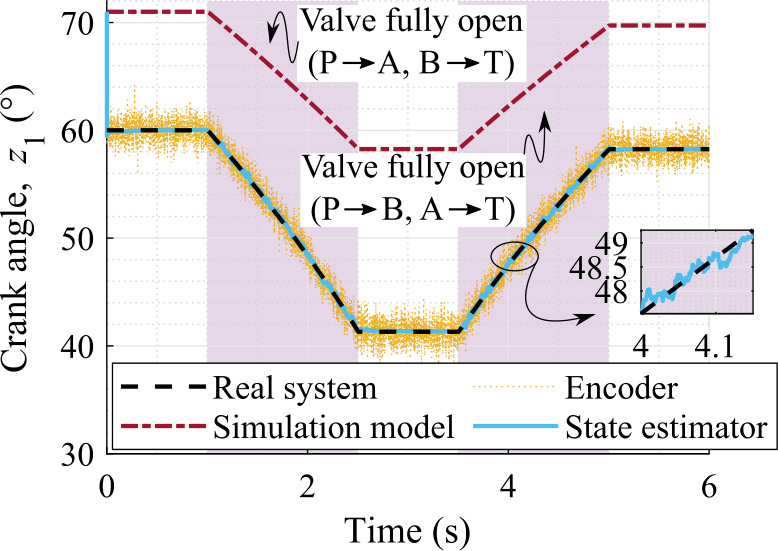
Comparison of the crank angle with sensor set-1 providing data at 1000 Hz

To better understand the behavior of the state estimator, the crank angle for the entire work cycle is shown in Fig. 7. Here, the hydraulic cylinder tilts the four-bar structure outwards between 1–2.5 s; holds it in this position between 2.5–3.5 s; tilts it inwards between 3.5–5 s; and holds it in this position to complete the work cycle. In all the subsequent figures, the hydraulic actuation regions are highlighted in purple. In Fig. 7, the RMSE of the crank angle is $0.23\%$ with respect to the absolute maximum value of the real system, at 1000 Hz sampling rate of the sensors. Whereas the RMSE of the encoder measurement is $1.67\%$. Thus, the state estimator provides a more accurate estimation of the crank angle than the encoder measurement, which is also evident from Fig. 7.

**Fig. 8:**
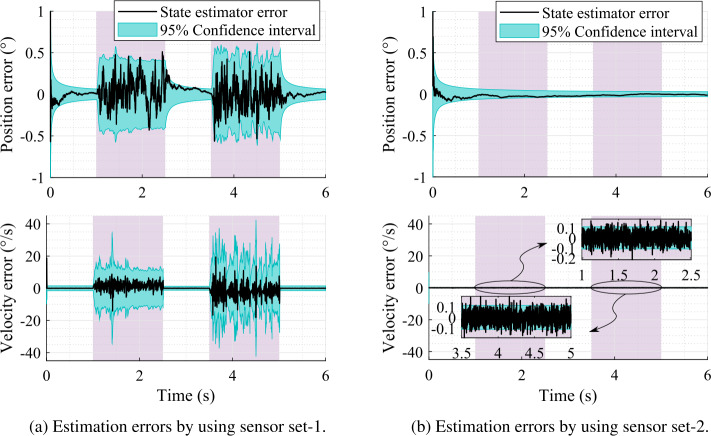
Position and velocity estimation errors with respect to the real system using sensors that provide data at 1000 Hz

Furthermore, the difference between the state estimator and the real system is almost indistinguishable in Fig. 7. Therefore, the actual position and velocity errors, that is, the crank angle and angular rate errors, with respect to the real system are shown in Fig. 8. Here the $95 \%$ confidence interval is consistent with the actual estimation errors, that is, it shrinks as the error decreases and grows as the error increases. Plots of this kind provide useful information about the system observability without the need for more formal observability analysis. Note that the $95 \%$ confidence interval is computed as $\pm 1.96 \sigma $, where $\sigma $ is the standard deviation calculated from the corresponding value of the covariance matrix, ${\mathbf{{P}}}^{+}$, associated with the state estimation.

In sensor set-1, the RMSE on the position and velocity levels are, respectively, $0.23\%$ and $12.02\%$ with respect to the absolute maximum value of the real system at 1000 Hz sampling rate of the sensors. Whereas, they are, respectively, $0.10\%$ and $0.28\%$ in case of sensor set-2 at 1000 Hz sampling rate. Thus, the overall estimation quality is improved by adding a velocity sensor, that is, a gyroscope, to a working sensor set, which is also shown in Fig. 8b. Note that the RMSE of the gyroscope measurement is $0.29\%$ in sensor set-2 at 1000 Hz sampling rate.

**Fig. 9:**
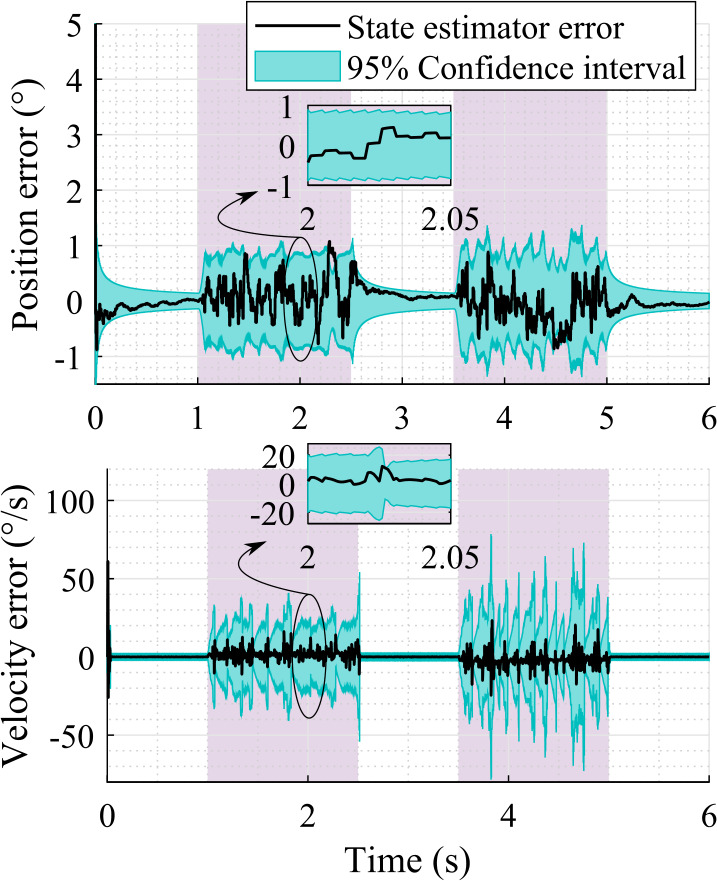
Position and velocity estimation errors with respect to the real system using sensor set-1 providing data at 200 Hz

Furthermore, Fig. 9 shows the position and velocity estimation errors when the sampling rate of the sensors is 200 Hz in sensor set-1. The saw-tooth shape of the $95 \%$ confidence interval shows the evolution of the covariance matrix, that is, the covariance grows when there is no measurement and shrinks when a measurement is available. The tracking error follows a similar trend.

### Accuracy of the hydraulic pressures estimation

**Fig. 10:**
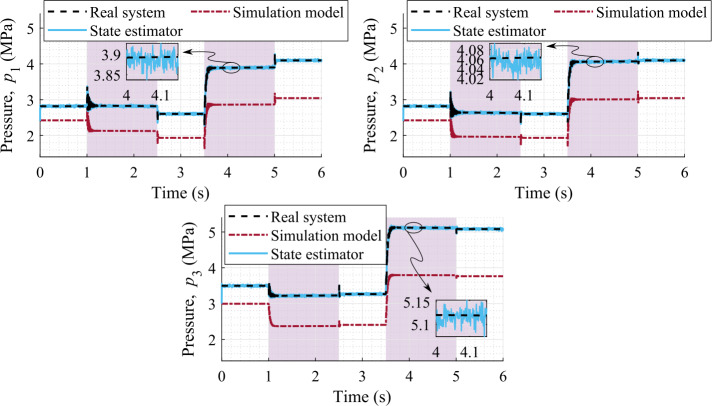
Comparison of hydraulic pressures with sensor set-1 providing data at 1000 Hz

**Fig. 11:**
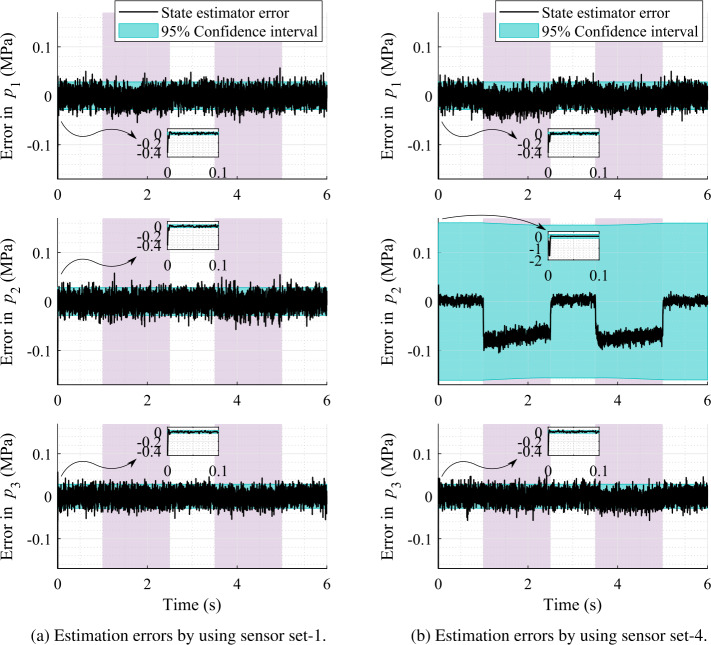
Pressure estimation errors with respect to the real system using sensors that provide data at 1000 Hz

The estimations of the hydraulic pressures are shown in Fig. 10, where the pressure estimation is almost indistinguishable from the real system. The RMSEs of the estimated pressures are, respectively, $0.34\%$, $0.33\%$, and $0.28\%$ with respect to the absolute maximum value of the real system, at 1000 Hz sampling rate of the sensors. Whereas, the RMSEs of the pressure sensors are, respectively, $0.35\%$, $0.34\%$, and $0.29\%$, at 1000 Hz sampling rate. Thus, the estimation of the hydraulic pressures is slightly improved in comparison with the pressure sensor measurements. Furthermore, for the plots in Fig. 10, the pressure errors are shown in Fig. 11a.

Note that in sensor set-1 (Fig. 10) the measurements for all pressures, $p_{1}$, $p_{2}$, and $p_{3}$, are used because they are assumed to be independent inside the filter. However, if an additional velocity sensor, that is a gyroscope, is used, then it enables one to remove one of the pressure sensors from either end of the hydraulic cylinder (Eq. (39)), such as for sensor sets 3 and 4. Otherwise, removing a pressure sensor without adding a velocity sensor would result either in a maximum number of iterations of the state estimator or a gradually increasing covariance of the pressure estimation. Note that the limit on the maximum number of iterations is defined within the implicit integrator used in this study. Moreover, the lack of convergence within the integrator is caused by the growth of the terms of the covariance matrix that leads to too large corrections, thus demonstrates the lack of observability. The pressure estimation error using sensor set-4 is shown in Fig. 11b.

**Fig. 12:**
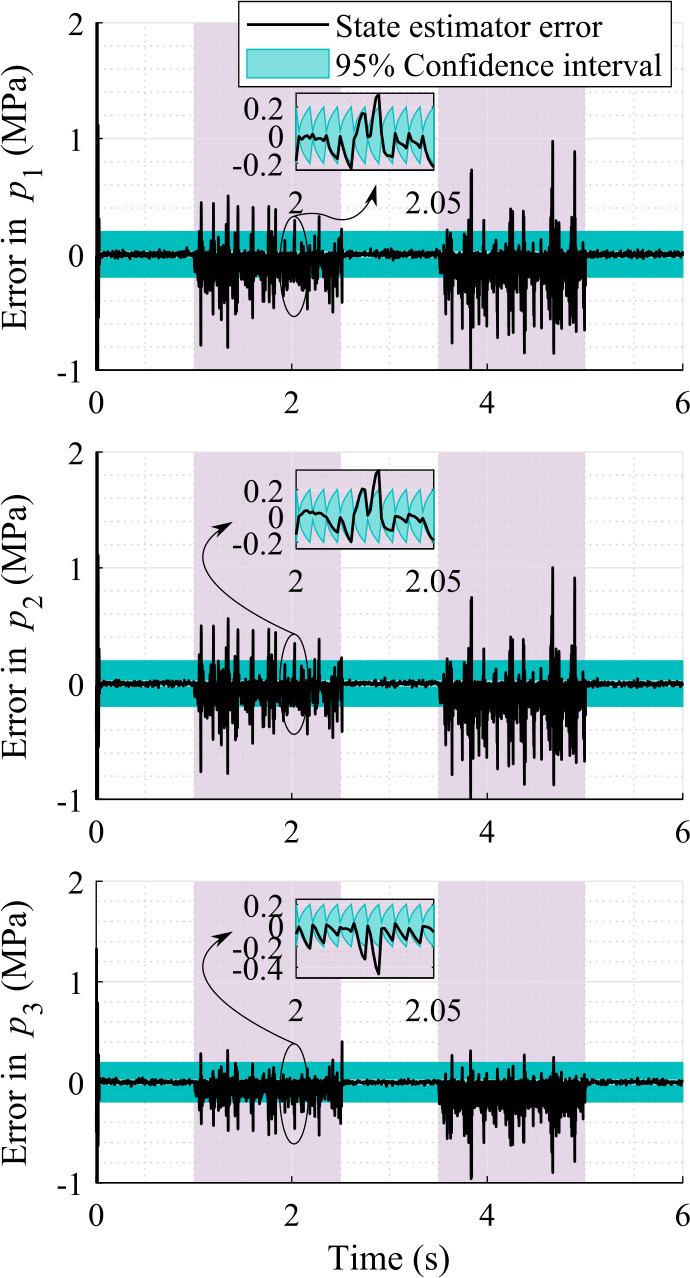
Pressure estimation errors with respect to the real system using sensor set-1 providing data at 200 Hz

At a lower sampling rate, 200 Hz, of the sensors, the pressure errors are shown in Fig. 12. At this sampling rate of the sensors, the $95 \%$ confidence interval of the pressure estimations follows a similar saw-tooth shape evolution as in the case of the position and velocity estimations.

## Conclusion

This paper introduced a novel state estimator developed by combining a multibody model and an indirect Kalman filter in the framework of hydraulically driven systems. An indirect Kalman filter that utilizes the exact Jacobian matrix of the state transition matrix at the position and velocity levels found in the literature has been extended for hydraulically actuated systems. The structures of the covariance matrices of the plant and measurement noise were also proposed. The multibody system was described using a semi-recursive formulation and the hydraulic subsystem using the lumped fluid theory. These two models were combined using a monolithic approach. The state estimator considered modeling errors in the force model, such as incorrect values of gravity and pump pressure, that affected the plant throughout the simulation. A non-formal demonstration of the system observability was provided by the introduction of errors in the initial values of position and pressure. The measurements in this study were obtained from a dynamic model, which was considered as the ground truth, with an addition of white Gaussian noise that represented the noise properties of the actual sensors.

The developed state estimator was illustrated on a hydraulically actuated four-bar mechanism using four sensor configurations with four different sampling rates. The state estimator provided a more accurate estimation, especially at the position level, compared to the measurements. Moreover, the confidence interval was consistent with the actual estimation errors. Furthermore, the overall estimation quality is improved by adding a velocity sensor, that is, a gyroscope, to a working sensor set. Moreover, the addition of a gyroscope enabled one to remove one of the pressure sensors from either end of the hydraulic cylinder, which is otherwise difficult under the given set of conditions. It has been observed that the accuracy of the estimation degrades when the sampling rate of the sensors is reduced. At a lower sampling rate, the evolution of the covariance matrix follows a saw-tooth shape, that is, the covariance grows when there is no measurement and shrinks when there is measurement available. The results demonstrated the efficacy of the proposed state estimator.

In future works, the proposed method will be tested on real world systems, such as a hydraulic crane, where the size of the system and its complexity are higher. The studies may also be directed towards reducing the number of pressure sensors, as the pressure build-up in the hydraulic sections is inter-dependent. The idea can be to protect the pressure sensors from damage caused by impacts from other parts of the working machinery in a limited working space. The estimation of hydraulic forces acting on a coupled multibody system will also be considered in future work.

## References

[1] Arulampalam M.S., Maskell S., Gordon N., Clapp T. (2002). A tutorial on particle filters for online nonlinear/non-gaussian Bayesian tracking. IEEE Trans. Signal Process..

[2] Bayo E., Jalon J.G.D., Serna M.A. (1988). A modified Lagrangian formulation for the dynamic analysis of constrained mechanical systems. Comput. Methods Appl. Mech. Eng..

[3] Bayo E., Ledesma R. (1996). Augmented Lagrangian and mass-orthogonal projection methods for constrained multibody dynamics. Nonlinear Dyn..

[4] Blanco-Claraco J.L., Torres-Moreno J.L., Giménez-Fernández A. (2015). Multibody dynamic systems as Bayesian networks: applications to robust state estimation of mechanisms. Multibody Syst. Dyn..

[5] Brown P., McPhee J. (2016). A continuous velocity-based friction model for dynamics and control with physically meaningful parameters. J. Comput. Nonlinear Dyn..

[6] Carpenter J., Clifford P., Fearnhead P. (1999). Improved particle filter for nonlinear problems. IEE Proc. Radar Sonar Navig..

[7] Cuadrado J., Cardenal J., Bayo E. (1997). Modeling and solution methods for efficient real-time simulation of multibody dynamics. Multibody Syst. Dyn..

[8] Cuadrado J., Cardenal J., Morer P., Bayo E. (2000). Intelligent simulation of multibody dynamics: space-state and descriptor methods in sequential and parallel computing environments. Multibody Syst. Dyn..

[9] Cuadrado J., Dopico D., Barreiro A., Delgado E. (2009). Real-time state observers based on multibody models and the extended Kalman filter. J. Mech. Sci. Technol..

[10] Cuadrado J., Dopico D., Gonzalez M., Naya M.Á. (2004). A combined penalty and recursive real-time formulation for multibody dynamics. J. Mech. Des..

[11] Cuadrado J., Dopico D., Naya M.Á., Gonzalez M. (2008). Real-time multibody dynamics and applications. Simulation Techniques for Applied Dynamics.

[12] De Geeter J., Van Brussel H., De Schutter J., Decréton M. (1997). A smoothly constrained Kalman filter. IEEE Trans. Pattern Anal. Mach. Intell..

[13] Grewal M.S., Andrews A.P. (2014). Kalman Filtering: Theory and Practice with MATLAB.

[14] Handroos H.M., Vilenius M.J. (1991). Flexible semi-empirical models for hydraulic flow control valves. J. Mech. Des..

[15] Jaiswal S., Islam M., Hannola L., Sopanen J., Mikkola A. (2018). Gamification procedure based on real-time multibody simulation. Int. Rev. Model. Simul..

[16] Jaiswal S., Korkealaakso P., Åman R., Sopanen J., Mikkola A. (2019). Deformable terrain model for the real-time multibody simulation of a tractor with a hydraulically driven front-loader. IEEE Access.

[17] Jaiswal S., Rahikainen J., Khadim Q., Sopanen J., Mikkola A. (2021). Comparing double-step and penalty-based semirecursive formulations for hydraulically actuated multibody systems in a monolithic approach. Multibody Syst. Dyn..

[18] Jaiswal S., Sopanen J., Mikkola A. (2021). Efficiency comparison of various friction models of a hydraulic cylinder in the framework of multibody system dynamics. Nonlinear Dyn..

[19] Jalon J.G.D., Alvarez E., Ribera F.A.D., Rodriguez I., Funes F.J. (2005). A fast and simple semi-recursive formulation for multi-rigid-body systems. Comput. Methods Appl. Sci..

[20] Jalon J.G.D., Bayo E. (1994). Kinematic and Dynamic Simulation of Multibody Systems: The Real-Time Challenge.

[21] Kalman R.E. (1960). A new approach to linear filtering and prediction problems. J. Basic Eng..

[22] Latif A., Chalhoub N., Pilipchuk V. (2020). Control of the nonlinear dynamics of a truck and trailer combination. Nonlinear Dyn..

[23] Linge S., Langtangen H.P. (2016). Programming for Computations-MATLAB/Octave: A Gentle Introduction to Numerical Simulations with MATLAB/Octave.

[24] Naya M.Á., Cuadrado J., Dopico D., Lugris U. (2011). An efficient unified method for the combined simulation of multibody and hydraulic dynamics: comparison with simplified and co-integration approaches. Arch. Mech. Eng..

[25] Pastorino R., Richiedei D., Cuadrado J., Trevisani A. (2013). State estimation using multibody models and non-linear Kalman filters. Int. J. Non-Linear Mech..

[26] Pastorino R., Sanjurjo E., Luaces A., Naya M.Á., Desmet W., Cuadrado J. (2015). Validation of a real-time multibody model for an x-by-wire vehicle prototype through field testing. J. Comput. Nonlinear Dyn..

[27] Potosakis N., Paraskevopoulos E., Natsiavas S. (2020). Application of an augmented Lagrangian approach to multibody systems with equality motion constraints. Nonlinear Dyn..

[28] Rahikainen J., Kiani M., Sopanen J., Jalali P., Mikkola A. (2018). Computationally efficient approach for simulation of multibody and hydraulic dynamics. Mech. Mach. Theory.

[29] Rahikainen J., Mikkola A., Sopanen J., Gerstmayr J. (2018). Combined semi-recursive formulation and lumped fluid method for monolithic simulation of multibody and hydraulic dynamics. Multibody Syst. Dyn..

[30] Sanjurjo, E.: State observers based on detailed multibody models applied to an automobile. Ph.D. thesis, University of A Coruña (2016)

[31] Sanjurjo E., Dopico D., Luaces A., Naya M.Á. (2018). State and force observers based on multibody models and the indirect Kalman filter. Mech. Syst. Signal Process..

[32] Sanjurjo E., Naya M.Á., Blanco-Claraco J.L., Torres-Moreno J.L., Giménez-Fernández A. (2017). Accuracy and efficiency comparison of various nonlinear Kalman filters applied to multibody models. Nonlinear Dyn..

[33] Simon D. (2006). Optimal State Estimation: Kalman, H Infinity, and Nonlinear Approaches.

[34] Simon D., Chia T.L. (2002). Kalman filtering with state equality constraints. IEEE Trans. Aerosp. Electron. Syst..

[35] Torres J., Blanco J., Sanjurjo E., Naya M.Á., Giménez A. (2014). Towards benchmarking of state estimators for multibody dynamics. The 3rd Joint International Conference on Multibody System Dynamics. the 7th Asian Conference on Multibody Dynamics.

[36] Van Loan C. (1978). Computing integrals involving the matrix exponential. IEEE Trans. Autom. Control.

[37] Watton J. (1989). Fluid Power Systems: Modeling, Simulation, Analog, and Microcomputer Control.

